# Illuminating the Live-Cell Dynamics of Hepatitis B Virus Covalently Closed Circular DNA Using the CRISPR-Tag System

**DOI:** 10.1128/mbio.03550-22

**Published:** 2023-02-22

**Authors:** Jiahui Ding, Zhigang Yi, Wenjing Zai, Min Wu, Baohui Chen, Qiliang Cai, Xiaonan Zhang, Zhenghong Yuan

**Affiliations:** a Key Laboratory of Medical Molecular Virology (MOE/NHC/CAMS), School of Basic Medical Sciences, Shanghai Medical College, Fudan University, Shanghai, China; b Shanghai Public Health Clinical Center, Fudan University, Shanghai, China; c Centre for Research in Therapeutic Solutions, Biomedical Sciences, Faculty of Science and Technology, University of Canberra, ACT, Australia; d Department of Cell Biology and Bone Marrow Transplantation Center of the First Affiliated Hospital, Zhejiang University School of Medicine, Hangzhou, China; University of Pittsburgh; McMaster University

**Keywords:** hepatitis b virus, CRISPR-Tag, cccDNA, live-cell imaging, minicircle

## Abstract

The covalently closed circular DNA (cccDNA) of hepatitis B virus (HBV) is the major obstacle to curing chronic hepatitis B (CHB). Current cccDNA detection methods are mostly based on biochemical extraction and bulk measurements. They nevertheless generated a general sketch of its biological features. However, an understanding of the spatiotemporal features of cccDNA is still lacking. To achieve this, we established a system combining CRISPR-Tag and recombinant HBV minicircle technology to visualize cccDNA at single-cell level in real time. Using this system, we found that the observed recombinant cccDNA (rcccDNA) correlated quantitatively with its active transcripts when a low to medium number of foci (<20) are present, but this correlation was lost in cells harboring high copy numbers (≥20) of rcccDNA. The disruption of HBx expression seems to displace cccDNA from the dCas9-accessible region, while HBx complementation restored the number of observable cccDNA foci. This indicated regulation of cccDNA accessibility by HBx. Second, observable HBV and duck HBV (DHBV) cccDNA molecules are substantially lost during cell division, and the remaining ones were distributed randomly to daughter cells. In contrast, Kaposi's sarcoma-associated herpesvirus (KSHV)-derived episomes can be retained in a LANA (latency-associated nuclear antigen)-dependent manner. Last, the dynamics of rcccDNA episomes in nuclei displayed confined diffusion at short time scales, with directional transport over longer time scales. In conclusion, this system enables the study of physiological kinetics of cccDNA at the single-cell level. The differential accessibility of rcccDNA to dCas9 under various physiological conditions may be exploited to elucidate the complex transcriptional and epigenetic regulation of the HBV minichromosome.

## INTRODUCTION

Despite the availability of an effective vaccine, there are still 240 million chronic carriers of hepatitis B virus (HBV) worldwide, according to a 2013 estimate (1). Patients with chronic hepatitis B (CHB) patients who do not undergo consistent antiviral treatment or medication compliance are at a significantly higher risk for developing liver fibrosis, cirrhosis, and hepatocellular carcinoma (HCC) (2). HBV is a member of the family *Hepadnaviridae*, which consists of small, hepatotropic DNA viruses and has a small, partially double-stranded genome. Following internalization of the viral particle, fusion of viral and cellular membranes, and transport of the capsid to nuclear pore, relaxed circular DNA (rcDNA) is released into the nuclei and converted into covalently closed circular DNA (cccDNA) (3). The cccDNA plays a central role in the HBV life cycle. It is maintained in the infected hepatocyte nuclei as a stable minichromosome and serves as the template for all HBV transcripts (4, 5). As cccDNA is notoriously refractory to commonly used nucleos(t)ide analogs (NAs) that suppress viral polymerase, (6, 7), it is widely regarded as the molecular basis for HBV persistence and the ultimate obstacle to developing an HBV cure.

Although the general biological characteristics of cccDNA have been elucidated (8–12), its spatiotemporal features within living cells are still unknown. It is generally believed that cccDNA is lost during cell division at the cell population level (13–15). However, the exact fate of cccDNA after one round of cell division at the single-cell level remains obscure. In addition, the dynamics of cccDNA within the nucleus is still largely unexplored. Traditional methods for cccDNA detection based on biological purification and bulk measurements, including Southern blotting and quantitative PCR (qPCR), restrict the study of the above-mentioned cccDNA spatiotemporal characteristics. The fluorescence *in situ* hybridization (FISH) assay provides additional spatial information on cccDNA (16–18) but is unable to track its live-cell dynamics.

Fortunately, noninvasive imaging techniques have been developed to study the spatiotemporal characteristics of DNA molecules. They have been applied to study the fate of host chromosomes during mitosis in live cell (19–21), the relationship between dynamics of gene loci and their transcriptional activity (21, 22), and the spatiotemporal organization of adenovirus replication (23). In particular, the development of a CRISPR/Cas9-mediated DNA imaging system has shown great promise. Initially, highly repetitive DNA loci can be visualized with a high signal-to-noise ratio (SNR), albeit with limited efficiency with nonrepetitive genomic sequences (19). More recently, an efficient CRISPR/Cas9-based DNA tag (1 kb) system, i.e., CRISPR-Tag, was developed with features of high versatility, scalability, and robustness (20).

Here, by leveraging the CRISPR-Tag system (20), we monitored the dynamics and fate of cccDNA in real time at the single-cell level. By exploiting the cccDNA minicircle technique (24–27), a recombinant HBV cccDNA with an insertion of a chimeric intron harboring CRISPR-Tag was prepared (HBV/duck HBV [DHBV] rcccDNA–CRISPR-Tag). This design rendered the minichromosome amenable to tracking while preserving the native viral replication and antigen expression as a result of the posttranscriptional splicing of the intron (28, 29). Using this system, the rcccDNA molecules were monitored with live-cell fluorescence microscopy. We carefully gauged the efficiency of this system by combining RNA/DNA FISH analysis and observed robust labeling efficiency when each nucleus harbored <20 copies of rcccDNA. We also found that the labeling efficiency of rcccDNA foci was affected by HBx expression, which may be related to the HBx-regulated accessibility of cccDNA. In addition, we observed that rcccDNA–CRISPR-Tag molecules were substantially lost in one-round cell division and the remaining molecules were randomly distributed to daughter cells. Finally, we describe confined diffusion accompanied by direct movement of cccDNA foci. These data support the utility of CRISPR-Tag in interrogating the intranuclear location and functional state of cccDNA.

## RESULTS

### Design of HBV rcccDNA–CRISPR-Tag tracking system.

**FIG 1:**
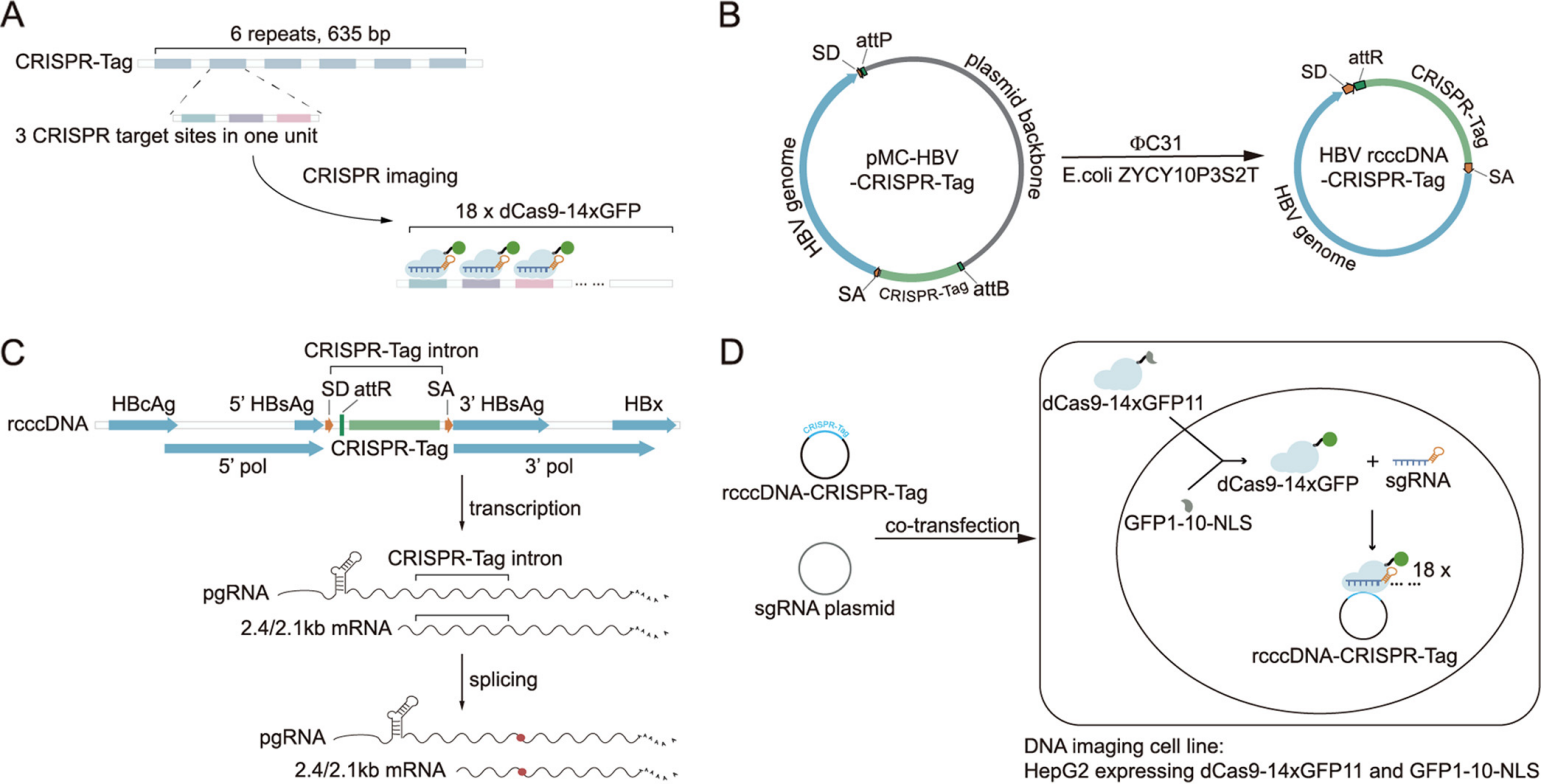
Schematic illustration of HBV cccDNA live-cell imaging strategy. (A) Schematic of CRISPR-Tag tagging system. (B) Schema of ϕC31 mediated recombination of parental plasmid (i.e., pMC-HBV-CRISPR-Tag) excising a HBV cccDNA episome with a CRISPR-Tag chimeric intron. SD, splicing donor site; SA, splicing acceptor site. (C) cccDNA sequence containing a CRISPR-Tag intron. Overlapping reading frames of the HBsAg and polymerase genes were interrupted by the insertion of an exogenous sequence between nucleotides 48 and 49 in the open reading frame of small HBs. The CRISPR-Tag intron was spliced from viral transcripts during RNA processing, without disturbing the replication cycle of HBV. (D) Overview of cccDNA imaging principle. rcccDNA–CRISPR-Tag and sgRNA plasmid were cotransfected to HepG2 cells expressing appropriate level of dCas9-14xGFP11 and GFP1-10-NLS. The dCas9-14xGFP11 and GFP1-10-NLS complementary to each other formed dCas9-14xGFP, which bound to HBV rcccDNA–CRISPR-Tag directed by sgRNA. A single copy of HBV rcccDNA–CRISPR-Tag was bound by multiple copies of dCas9-14xGFP to generate a detectable spot using fluorescence microscopy.

To monitor the cccDNA in real time, we devised a tracking system based on the CRISPR-Tag technology. The 635-bp CRISPR-Tag contains six repeats, each of which harbors three prevalidated single guide RNA (sgRNA) target sites originating from the Caenorhabditis elegans genome (20) (Fig. 1A). Using the DNA sequence from C. elegans avoided unspecific labeling in human cells. Multiple binding sites for dCas9 improved detection sensitivity (Fig. 1A). In order to preserve the native viral replication cycle, the CRISPR-Tag was flanked by a pair of splicing donor (SD) and splicing acceptor (SA) sites (28, 29), such that the mature pregenomic RNA and other messenger RNAs would be devoid of the tag (Fig. 1B and C). As the rcDNA was free of the tag, the specificity of the cccDNA labeling was ensured (Fig. 1C). In addition, the minicircle technique was used to prepare recombinant cccDNA devoid of the bacterial backbone (24–27) (Fig. 1B). The CRISPR-tagged HBV genome was flanked by two recombination sites, attB and attP, and inserted into a prokaryotic plasmid to generate the construct, i.e., pMC-HBV-CRISPR-Tag (Fig. 1B, left). In a minicircle producer cell expressing phage ϕC31 integrase, the recombinant cccDNA (HBV rcccDNA–CRISPR-Tag) was excised by integrase-mediated intramolecular recombination, resulting in a circular DNA containing an attR site and the CRISPR-Tag within the chimeric intron (Fig. 1B, right). As a result, the CRISPR-Tag flanked by a pair of SD site and SA site were inserted after nucleotide (nt) 48 of HBV small surface antigen (HBsAg) open reading frame (subtype ayw; GenBank accession no. V01460.1) (Fig. 1B and C, top). A residual attR sequence as the result of the DNA recombination remained between the SD site and the CRISPR-Tag (Fig. 1B, right; Fig. 1C). After transcription, all exogenous sequences were spliced out, and the mature viral mRNAs were identical to those from the wild-type virus (Fig. 1C).

Another key component of our live-cell cccDNA tracking system is a cell line stably expressing dCas9-guided fluorescent proteins (Fig. 1D). We adopted the tandem split green fluorescent protein (GFP) system to amplify the fluorescence (20). Specifically, a repeating array containing 14 copies of GFP11 was fused to dCas9 (dCas9-14xGFP11), which can recruit sgRNA to assemble a guided complex that binds to the CRISPR-Tag sequence. As a result, up to 252 complemented GFP molecules could bind to a single copy of HBV rcccDNA, thus enabling easy detection using fluorescence microscopy (Fig. 1D).

### Validation of the rcccDNA–CRISPR-Tag tracking system.

Following the strategy mentioned above, we constructed and validated the CRISPR-tagged rcccDNA tracking system.

**FIG 2:**
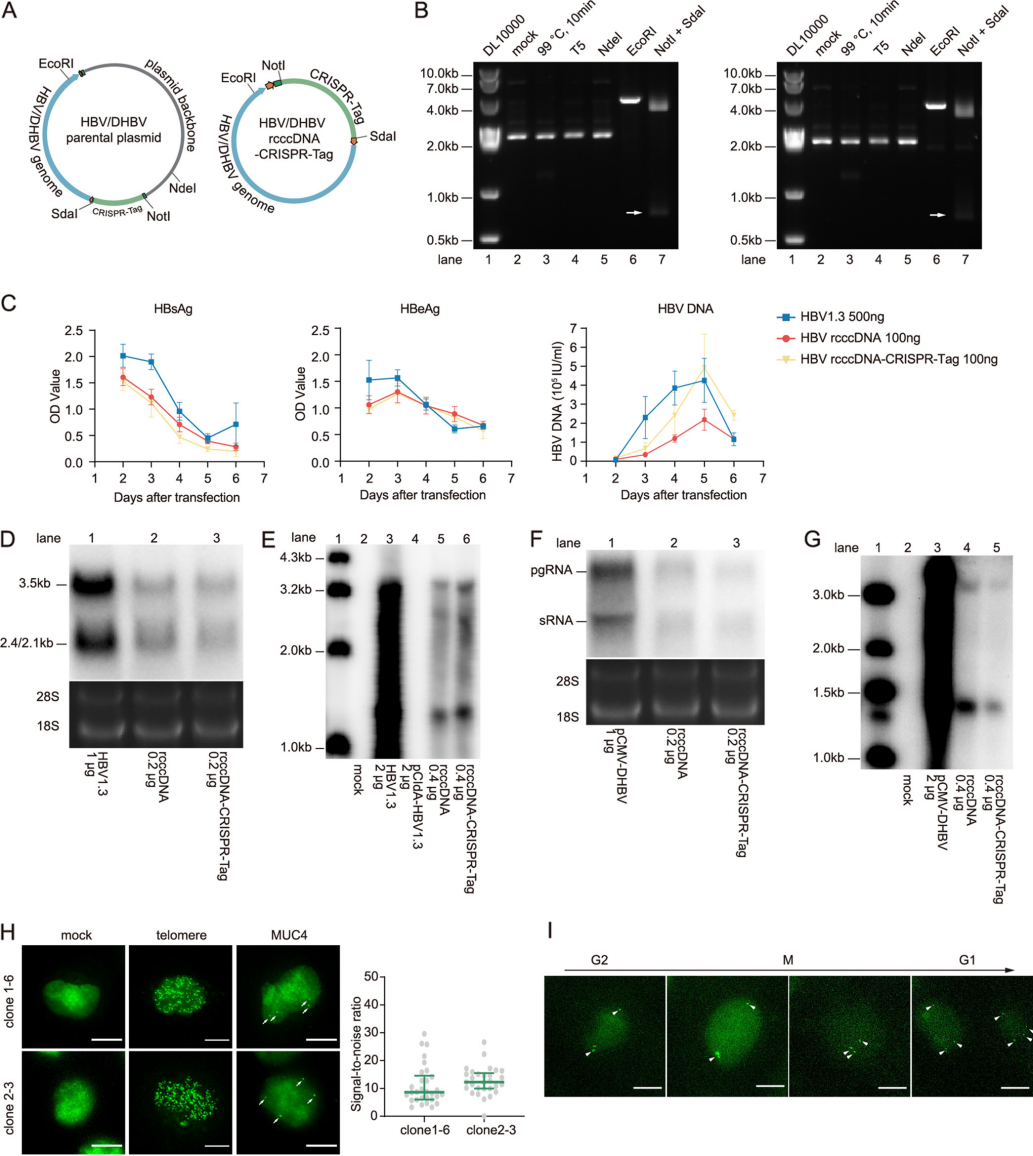
Construction of the rcccDNA–CRISPR-Tag live-cell imaging system. (A) The sites of restriction endonucleases used to identify parental plasmid and rcccDNA–CRISPR-Tag are shown. (B) Production of HBV and DHBV rcccDNA–CRISPR-Tag. After minicircle production, HBV (left) and DHBV (right) rcccDNA–CRISPR-Tag was denatured by heat treatment at 99°C and digested with T5 exonuclease, EcoRI, NdeI, NotI, and SdaI. Results of electrophoresis analysis are shown. (C to E) Verification of rcccDNA–CRISPR-Tag-dependent HBV transcription, expression, and replication. HBV1.3, HBV rcccDNA, and HBV rcccDNA–CRISPR-Tag were transfected into DI-3 cells, and HBsAg, HBeAg, and HBV DNA (C), HBV transcripts (D), and HBV replication (E) were analyzed. Data are means and standard deviations. (F and G) Verification of rcccDNA–CRISPR-Tag-dependent DHBV transcription and replication. pCMV-DHBV, DHBV rcccDNA, or DHBV rcccDNA–CRISPR-Tag were transfected to DI-3 cells, DHBV transcripts (F) and replication (G) were detected. (H) Construction a HepG2-derived cell clone supporting single-molecule DNA detection. CRISPR imaging of MUC4 loci and telomere loci in selected cell clones. Labeling efficiency of MUC4 loci was determined by quantifying signal-to-noise ratio (*n* > 25 cells). The green line denotes the median with the interquartile range. (I) Snapshots of MUC4 loci in which a DI-3 cell undergoes mitosis, showing *z* maximum projections of 10 μm depth. The arrows indicate the MUC4 loci, which are not completely captured during mitosis because the cell thickness exceeds the *z* range. Bars, 10 μm.

First, we generated HBV and DHBV rcccDNA–CRISPR-Tag. We verified the minicircles by restriction enzyme digestion (Fig. 2A and B) and by Sanger sequencing (data not shown). We chose enzymes with restriction sites on the plasmid backbone (NdeI), the HBV and DHBV genome (EcoRI), and flanked CRISPR-Tag (SdaI and NotI) (Fig. 2A). The electrophoresis of HBV and DHBV rcccDNA–CRISPR-Tag showed a major band around 2.0 kb accompanied by a minor band, which was most probably the relaxed circular form (Fig. 2B, lane 2). Its resistance to T5 exonuclease digestion and 99°C heating confirmed its supercoiled nature (Fig. 2B, lanes 3 and 4). The removal of the plasmid backbone was confirmed by its resistance to NdeI digestion (Fig. 2B, lane 5). After EcoRI linearization, the HBV and DHBV rcccDNA–CRISPR-Tag band shifted to a double-stranded linear (DSL) position at around 4 kb (Fig. 2B, lane 6). The integrity of CRISPR-Tag was demonstrated by digestion with NotI and SdaI, which led to a band below 1.0 kb consistent with the length of CRISPR-Tag (Fig. 2B, lane 7, arrow). These data confirmed the proper insertion of CRISPR-Tag into the recombinant cccDNA.

We went on to test the transcription, replication and antigen expression mediated by HBV rcccDNA–CRISPR-Tag. The insertion of CRISPR-Tag did not compromise antigen secretion (Fig. 2C; compare HBV rcccDNA [red] with HBV rcccDNA–CRISPR-Tag [yellow]). Northern blot analysis demonstrated that HBV RNAs generated from HBV rcccDNA–CRISPR-Tag exhibited an electrophoretic pattern identical to that of the wild-type HBV RNAs, indicating correct splicing of CRISPR-Tag (Fig. 2D). The transcription level of HBV rcccDNA–CRISPR-Tag was slightly lower than that of HBV rcccDNA, indicating that splicing marginally affects the rate of HBV RNA synthesis (Fig. 2D). The detection of core particle-associated HBV DNA indicated replication competence of HBV rcccDNA–CRISPR-Tag. The detected signal was indistinguishable from or slightly stronger than that from HBV rcccDNA (Fig. 2E, compare lane 5 with lane 6). In comparison, pCIdA-HBV (30), which harbors 5′ ε RNA signal (packaging signal) deletion, did not generate any signal (Fig. 2E, lane 4). According to HBV DNA quantification results, HBV rcccDNA–CRISPR-Tag produced a higher level of HBV DNA than HBV rcccDNA (*P* = 0.0247, day 5) (Fig. 2C). Similarly, we compared the transcription and replication initiated by DHBV rcccDNA and DHBV rcccDNA–CRISPR-Tag. CRISPR-Tag insertion largely preserved DHBV transcript production and genome replication, albeit at slightly lower level (Fig. 2F and G). Thus, we validated the capacity of the CRISPR-tagged recombinant cccDNA to initiate a typical viral life cycle.

We then sought to establish a cell model to illuminate CRISPR-tagged rcccDNA for live-cell imaging. After transduction of lentiviruses encoding dCas9-14xGFP11 and GFP1-10-NLS into HepG2 cells, single-cell clones were isolated and tested for their imaging performance. These clones were transduced with lentivirus expressing an sgRNA-targeting telomere or MUC4 (mucin4) and observed under a fluorescence microscope as described in a previous report (19). Of a total of 73 clones, two displayed clear labeling of telomere and MUC4 loci (Fig. 2H). Four MUC4 loci were observed in HepG2-derived cells, suggesting that our cell clone was capable of single-particle tracking (Fig. 2H, panel 3). We finally selected clone 2-3 based on its higher SNR and cellular uniformity (Fig. 2H). It was termed DNA imaging cell clone 3 (DI-3) in subsequent experiments. Using DI-3, we observed the dynamics of the MUC4 loci during mitosis. These loci were observed to duplicate themselves, localize at the equatorial plate during metaphase, and separate into two daughter cells equally (Fig. 2I). The successful tracking of MUC4 replication and distribution during mitosis indicated that DI-3 supported the observation of a single gene locus.

### Live-cell imaging of CRISPR-tagged recombinant cccDNA.

**FIG 3:**
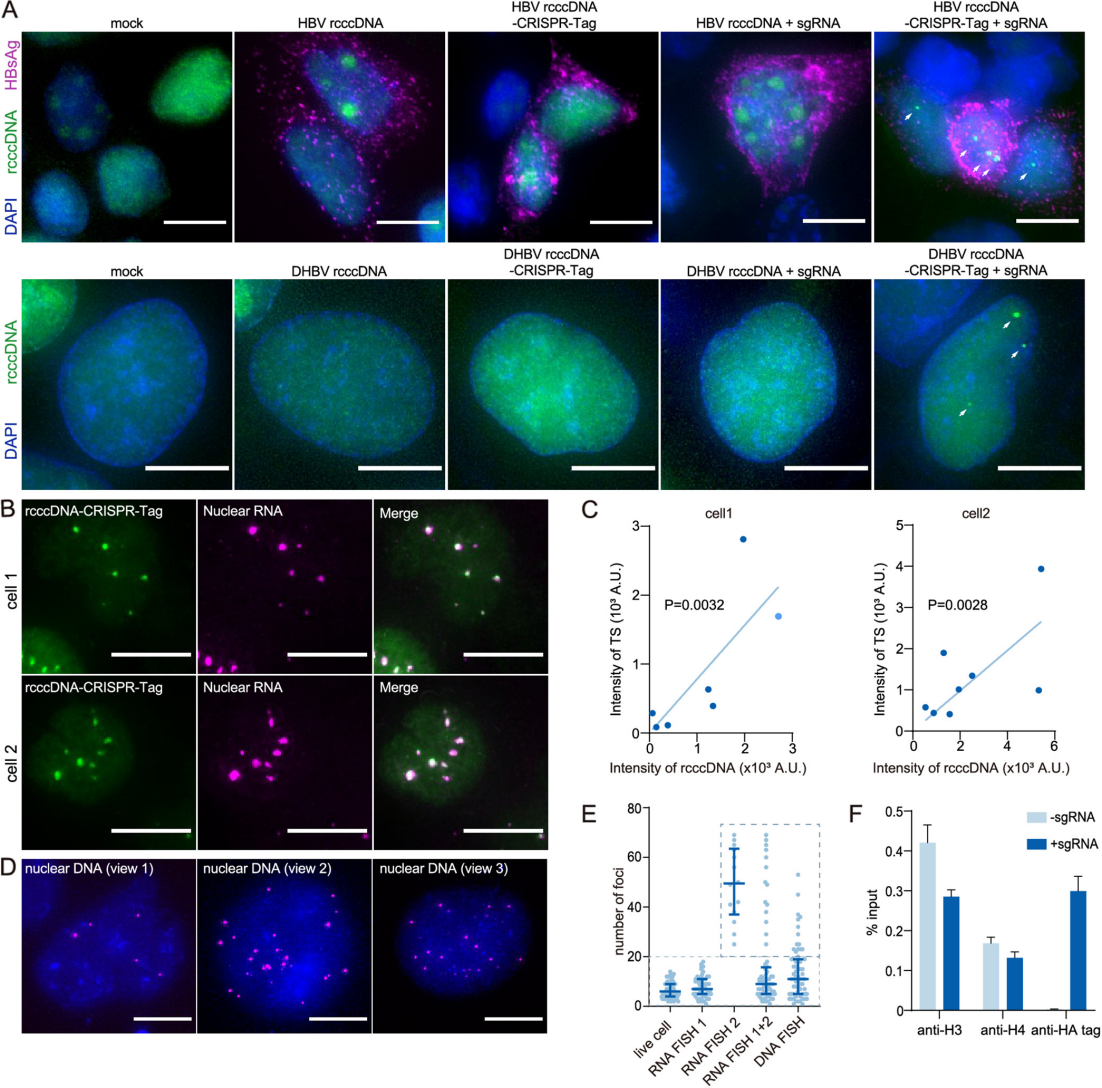
Detection of rcccDNA–CRISPR-Tag using the CRISPR/Cas9 system. DI-3 cells were cotransfected with HBV/DHBV rcccDNA/rcccDNA–CRISPR-Tag (50 ng) and pcDNA3.1/sgRNA plasmid (450 ng). (A) HBV (top) and DHBV (bottom) rcccDNA loci labeled by CRISPR imaging. Two days after transfection, cells were fixed and HBsAg was detected by immunofluorescence. The arrows indicate the rcccDNA–CRISPR-Tag foci. (B) Transcription sites of HBV rcccDNA–CRISPR-Tag detected by nuclear RNA FISH. Cells were fixed and stained with probe targeting the CRISPR-Tag (intron region) sequence. Foci labeled by CRISPR imaging and RNA FISH were observed. (C) Correlation between CRISPR focus intensity and RNA focus intensity in panel B. (D) Detection of HBV rcccDNA–CRISPR-Tag with a bDNA-based FISH assay. Two days after transfection, nuclei were prepared and stained with an HBV probe set. (E) Numbers of HBV rcccDNA–CRISPR-Tag foci detected by different methods. The numbers of CRISPR imaging foci (live cell, 58 cells), transcription sites in cells with high-SNR CRISPR foci (<20 foci, RNA FISH 1, 58 cells), transcription sites in cells without high-SNR CRISPR foci (≥20 foci, RNA FISH 2, 14 cells), transcription sites in both cell populations (RNA FISH 1+2, 72 cells), and fluorescent spots detected by bDNA-based DNA FISH (DNA FISH, 65 cells) are displayed. The box shows the overlap in population counts among groups. Data are medians and interquartile ranges. (F) Labeling efficiency of CRISPR imaging detected by ChIP assay. The ChIP assay was performed with antibodies specific for histone 3, histone 4, and the HA tag (fusing with dCas9). HBV rcccDNA–CRISPR-Tag was quantified by qPCR and normalized to an aliquot of the total input. Data are means and standard deviations. Bars, 10 μm.

We then attempted to detect HBV rcccDNA–CRISPR-Tag within DI-3 cells. Without sgRNA or CRISPR-Tag, neither rcccDNA nor rcccDNA–CRISPR-Tag can generate any fluorescent foci (Fig. 3A, second, third, and fourth panels). In contrast, with the cointroduction of CRISPR-Tag-specific sgRNAs, rcccDNA–CRISPR-Tag generated discernible foci which coincided with HBsAg positive cells (Fig. 3A, last panel). This suggested that these positive foci were bona fide cccDNA signals. To further confirm the labeling specificity of CRISPR imaging, we colabeled rcccDNA loci with transcription sites (TS) by RNA FISH using CRISPR-Tag (intron region) specific probes. The specificity of TS detection was confirmed by treatment with actinomycin D, RNase A, and DNase I (see Fig. S1A in the supplemental material). The RNA focus number was reduced after the treatments with actinomycin D and RNase A, while DNase I treatment did not affect RNA focus number (Fig. S1B). CRISPR focus signals generated by GFP proteins were affected by neither of the nuclease treatments postfixation (Fig. S1B).

10.1128/mbio.03550-22.1 FIG S1 Validation of HBV nuclear DNA and RNA FISH assay (see Fig. 3). (A) Transcription sites of rcccDNA–CRISPR-Tag were detected by nuclear RNA FISH. The specificity was validated by treating the sample with 4 μg/mL actinomycin D for 3 h prior to fixation in paraformaldehyde, 100 μg/mL RNase A 1 h after fixation, and 50 U/mL DNase I after fixation. (B) Quantification of the CRISPR foci and the RNA foci numbers in (A). (C) bDNA-based DNA FISH assay to detect cccDNA before (left) and after (right) nuclei isolation. (D) Transcription sites in a cell without high SNR CRISPR focus detection. Bar, 10 μm. Download FIG S1, TIF file, 2.2 MB. Copyright © 2023 Ding et al. 2023 Ding et al. https://creativecommons.org/licenses/by/4.0/ This content is distributed under the terms of the Creative Commons Attribution 4.0 International license.

We observed that the TS were uniformly colocalized with CRISPR foci when a low to medium number of copies of rcccDNA (<20 foci) was present (Fig. 3B). We calculated the number of foci detected by different methods. CRISPR focus number was consistent with TS number in the same cell (Fig. 3E, “live cell” and “RNA FISH 1”), and the intensity of each dot was also positively correlated (Fig. 3C). However, such colocalization was less evident when a high number of TS sites per cell (≥20 foci) were present (Fig. 3E, “RNA FISH 2”; Fig. S1D). We also detected the rcccDNA–CRISPR-Tag by combining the previously developed branched-DNA (bDNA)-based FISH method (17) and the nucleus isolation method (16), which may be less affected by the issues related to live-cell imaging (Fig. 3D; Fig. S1C). The resulting statistics indicated a higher median copy number than that seen with the CRISPR-Tag method (Fig. 3E). Interestingly, the CRISPR-Tag consistently failed to detect nuclear cccDNA with more than 20 copies of RNA foci, which corresponds to the upper 25% of the DNA FISH^+^ cells. These data indicated the limited detectability of a high copy number of rcccDNA using CRISPR-Tag, while a higher chance of detection can be achieved with low to medium copy numbers of rcccDNA under active transcription. It is worth noting that we were unable to colocalize the CRISPR-Tag and DNA FISH signal due to the quenching of GFP signal by some steps of the DNA FISH protocol.

In addition, a chromatin immunoprecipitation (ChIP) assay with antibody specific to the hemagglutinin (HA) tag (fused to dCas9) showed that dCas9 efficiently bound to rcccDNA–CRISPR-Tag, while no binding of dCas9 was detected in the absence of sgRNAs (Fig. 3F). Quantification of ChIP-captured viral DNA suggested that dCas9 was efficiently recruited to cccDNA upon specific sgRNAs (Fig. 3F). Above data showed rcccDNA–CRISPR-Tag was efficiently labeled by CRISPR imaging.

Furthermore, we evaluated the effects of CRISPR-imaging on characteristics of rcccDNA. The epigenetic modifications of H4ac, H3ac, H3K9me3, H3K4me3, and H3K27me3 were all detected (Fig. S2B) and showed no substantial difference from that of infection or rcccDNA–CRISPR-Tag without CRISPR labeling. The distribution of these epigenetic modifications measured by ChIP-seq was not significantly affected by CRISPR imaging (Fig. S2A). The nucleosome distribution measured by micrococcal nuclease digestion with deep sequencing (MNase-seq) was not significantly different from that seen with cccDNA in the infection system and rcccDNA–CRISPR-Tag without labeling (Fig. S2C). The transcription level, antigen expression level, and transcript distribution were not obviously compromised by CRISPR imaging (Fig. S2D to F). Thus, CRISPR imaging would not significantly affect the characteristics of rcccDNA–CRISPR-Tag.

10.1128/mbio.03550-22.2 FIG S2 Effects of CRISPR imaging on the biological characteristics of rcccDNA–CRISPR-Tag (see Fig. 3). HBV rcccDNA–CRISPR-Tag was transfected into DI-3 with or without sgRNA plasmid, respectively. (A and B) Epigenetic modifications of dCas9-binding HBV rcccDNA–CRISPR-Tag. ChIP assays were performed with the indicated antibodies. The distribution of posttranslational modifications was determined by ChIP-seq assay (A). Posttranslational modifications were quantified by ChIP-PCR assay (B). (C) Distribution of nucleosomes along rcccDNA–CRISPR-Tag. Mononucleosomal HBV DNAs obtained from 50-U/mL MNase digestion were analyzed by high-throughput sequencing, and the reads were aligned to HBV cccDNA. (D) HBV antigen level was detected by ELISA. (E and F) HBV transcript analysis by Northern blotting (E) and transcriptome sequencing (RNA-seq) (F). Data are means and SD. Relative read density for each track is represented by height on the *y* axis. HBV transcripts are represented on the *x* axis. Download FIG S2, TIF file, 1.1 MB. Copyright © 2023 Ding et al. 2023 Ding et al. https://creativecommons.org/licenses/by/4.0/ This content is distributed under the terms of the Creative Commons Attribution 4.0 International license.

Collectively, these data indicated that the CRISPR-Tag system can reliably detect transcription-competent cccDNA when a low to medium number of copies is present within cells.

### Characteristics of rcccDNA–CRISPR-Tag foci regulated by HBx and HBc proteins.

As the characteristics of CRISPR foci may be related to the molecular state of the particular gene loci, such as transcription activity and DNA damage and repair (31–33), considering that HBx regulated the transcription and accessibility of cccDNA (34–38) and HBc can bind to cccDNA to regulate its characteristics (39–41), we sought to explore the characteristics of rcccDNA–CRISPR-Tag foci regulated by HBx and HBc.

**FIG 4:**
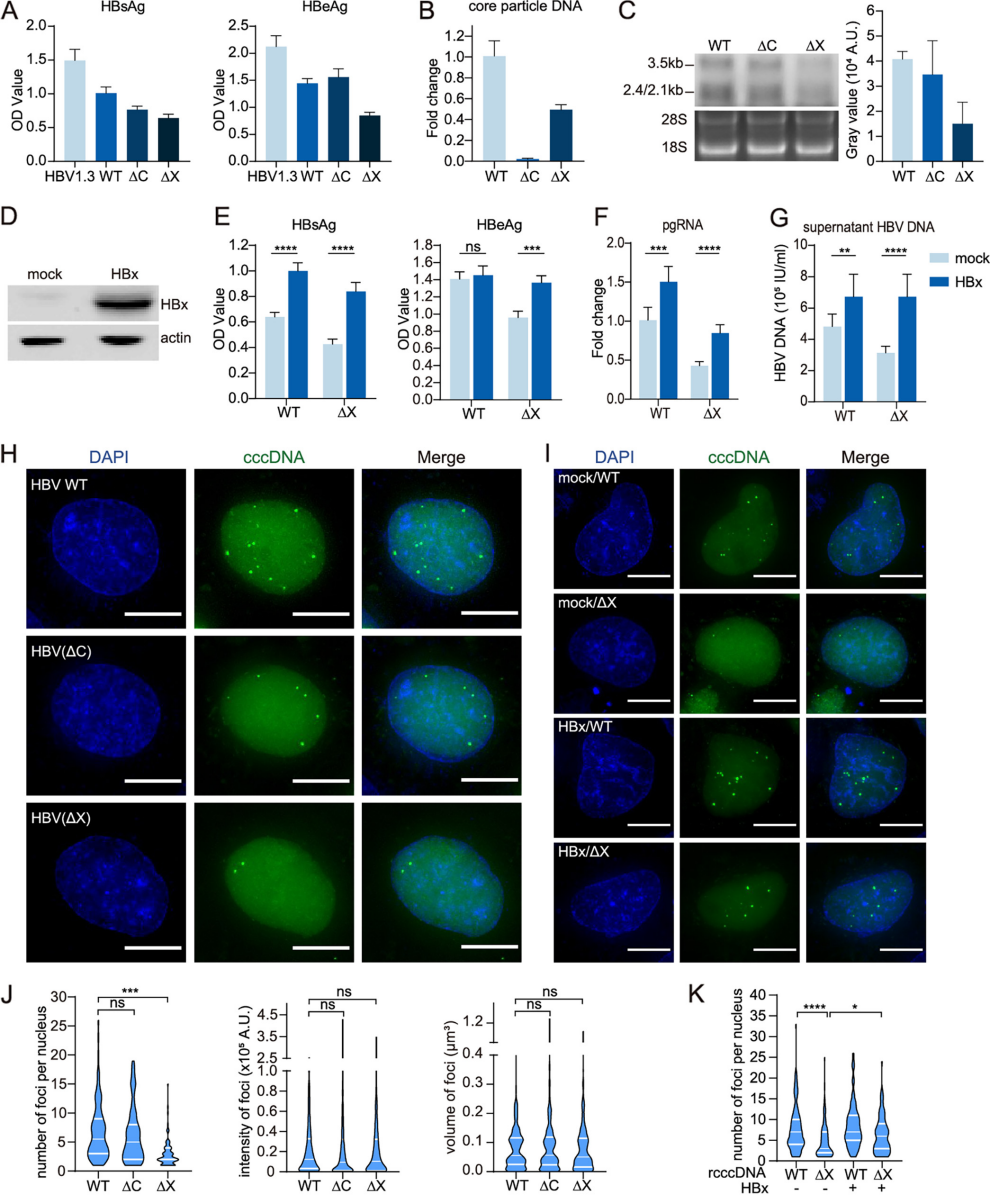
Comparison of foci among wild-type, HBc-null, and HBx-null rcccDNA–CRISPR-Tag constructs. (A to C) Validation of the construction of HBc-null (ΔC) and HBx-null (ΔX) rcccDNA–CRISPR-Tag. DI-3 cells were transfected with wild-type (WT), ΔC, and ΔX rcccDNA–CRISPR-Tag, culture supernatant was collected, and HBV antigens (A), cytoplasm core particle-associated DNA (B), and HBV transcripts were detected (C). (D) Validation of the expression of HBx in DI-3 cells. HBx-Flag was stably expressed in DI-3 cells using lentivirus, and its expression was detected by Western blotting using antibody specific for the Flag tag. (E to G) Validation of the function of HBx expressed in DI-3 cells. WT or ΔX rcccDNA–CRISPR-Tag was transfected into DI-3 cells with or without HBx overexpression. HBV antigens (E), pgRNA (F), and supernatant HBV DNA (G) were measured. (H) Representative snapshots of WT, ΔC, and ΔX rcccDNA–CRISPR-Tag foci in DI-3 cells. (I) Representative snapshots of WT and ΔX rcccDNA–CRISPR-Tag foci in DI-3 cells with or without HBx overexpression. (J) The number per nucleus, intensity, and volume of foci in (H) were quantified (*n* ≥ 94 cells). (K) The numbers of foci per nucleus in panel I were quantified (*n* ≥ 88 cells). Bars, 10 μm. Data are means and standard deviations. White lines represent medians, with interquartile ranges. *, *P* < 0.05; **, *P* < 0.01; ***, *P* < 0.001; ****, *P* < 0.0001; ns, not significant.

To test whether HBx and HBc can affect the characteristics of rcccDNA foci, we constructed HBc-null (ΔC) and HBx-null (ΔX) rcccDNA–CRISPR-Tag. Complete blockage of core particle DNA formation by HBc-null constructs and significant reduction of HBV transcripts and antigen by HBx-null constructs verified successful mutation construction (Fig. 4A to C). Then, we surveyed the characteristics (number, intensity, and volume) of HBV and HBV mutant rcccDNA–CRISPR-Tag foci (Fig. 4H and J). Interestingly, HBV(ΔX) (2 foci per nucleus, median of foci, *n* = 99 cells) caused many fewer foci in the nucleus than wild-type (WT) HBV (5.5 foci per nucleus, median of foci, *n* = 106 cells) and HBV(ΔC) (5 foci per nucleus, median of foci, *n* = 94 cells) (*P* < 0.0001 compared ΔX with WT and ΔC, Student’s unpaired two-tailed *t* test), while their intensity and volume appeared not to be affected (Fig. 4H and J). HBV(ΔC) did not significantly affect any major characteristics of foci. In addition, HBx expression in *trans* restored the compromised number of foci caused by HBV(ΔX) (Fig. 4D to G, I, and K). Since the disruption of HBx or HBc expression does not affect the copy number of rcccDNA, the dependence of CRISPR-Tag imaging on HBx expression seems to suggest that HBx altered the transcriptional state and the accessibility of dCas9-GFP. Thus, the CRISPR-Tag imaging system for cccDNA might serve as a useful indicator of intranuclear cccDNA accessibility.

Overall, the characteristics of rcccDNA–CRISPR-Tag foci could be affected by HBx but not HBc proteins, suggesting that decreasing labeling efficiency and capability of dCas9 binding may be related to a lower accessibility and transcription activity of cccDNA as a result of HBx inactivation.

### Dynamics of CRISPR-tagged cccDNA during mitosis.

**FIG 5:**
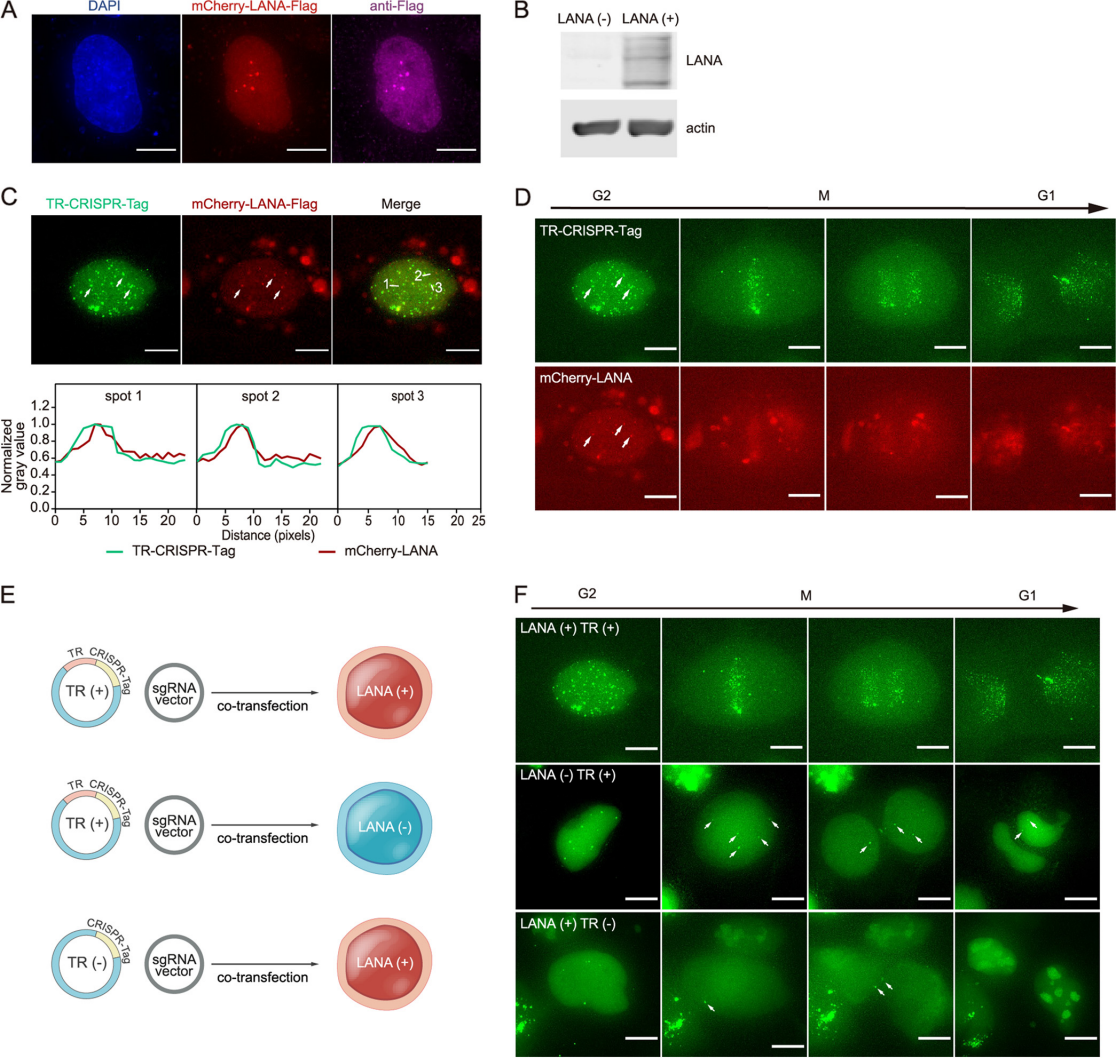
Dynamics of TR sequence-containing episomes through mitosis. (A and B) Verification of mCherry-LANA expression in DI-3 cells using immunofluorescence (A) and Western blotting (B). (C) Colocalization between TR–CRISPR-Tag episome loci and mCherry-LANA puncta. A representative image (top) and plot profile of three colocalized puncta (arrows) (bottom) are shown. (D) TR–CRISPR-Tag episome foci and mCherry-LANA puncta colocalized through mitosis. See Movie S1. (E) Experiment design for observing the dynamics of TR-containing episomes through mitosis (F). A plasmid containing KSHV TR sequence or CRISPR-Tag was transfected into DI-3 cells with or without mCherry-LANA expression. The fluorescent foci were observed using time-lapse microscopy. (F) Snapshots of different episome image sequences in which DI-3 cells with or without LANA expression underwent mitosis. See Movie S1. Bars, 10 μm.

Next, we used this system to track the dynamics of rcccDNA during mitosis. To test whether our system can truly reflect the fate of episome through mitosis, we introduced an episome containing the Kaposi's sarcoma-associated herpesvirus (KSHV) terminal repeat (TR) and CRISPR-Tag (TR–CRISPR-Tag). It has been widely recognized that LANA (latency-associated nuclear antigen) protein of KSHV supports the replication and persistence of a TR sequence-containing episome during mitosis (42, 43). Hence, we followed the dynamics of TR^−^ and TR^+^ episomes in LANA^−^ and LANA^+^ cells (Fig. 5E). The expression of LANA was confirmed by immunofluorescence and Western blotting (Fig. 5A and B). The TR–CRISPR-Tag foci colocalized with LANA puncta through mitosis (Fig. 5C and D; Movie S1). Compared with TR^−^ or LANA^−^ groups (Fig. 5E and F; Movie S1), more TR–CRISPR-Tag foci and their distribution to daughter cells in TR^+^ LANA^+^ group (Fig. 5E and F; Movie S1) implied that TR–CRISPR-Tag episomes were effectively amplified and maintained with the assistance of LANA (43, 44). Collectively, our live-cell imaging platform faithfully reflected the dynamics of TR–CRISPR-Tag through mitosis.

10.1128/mbio.03550-22.6 MOVIE S1 Dynamics of TR-sequence containing episomes through mitosis (see Fig. 5D and F). The loss and distribution of TR^−^ and TR^+^ episomes in LANA^−^ and LANA^+^ DI-3 cells undergoing mitosis were observed. LANA puncta (red) and TR sequence-containing episomes (green) were simultaneously displayed in the LANA^+^ TR^+^ group. The video was acquired as a *z* stack at a 0.5-μm step size and with a total of 20 steps. One *z* stack was taken every 15 min. *z* projection with a maximum-intensity merge signal is shown. Bar, 10 μm. Download Movie S1, AVI file, 11.2 MB. Copyright © 2023 Ding et al. 2023 Ding et al. https://creativecommons.org/licenses/by/4.0/ This content is distributed under the terms of the Creative Commons Attribution 4.0 International license.

We then used this system to track the dynamics of HBV and DHBV rcccDNA during mitosis. In order to capture mitosis events more efficiently for statistics, we introduced a reported fluorescent cell cycle indicator (45), in which S/G_2_/M phase was labeled by mCherry-hGeminin(1–110) (Fig. S3A and S3C; Movie S2). The expression of mCherry-hGeminin(1–110) did not disturb the normal cell cycle, as analyzed by flow cytometry and live-cell imaging (Fig. S3B and C).

**FIG 6:**
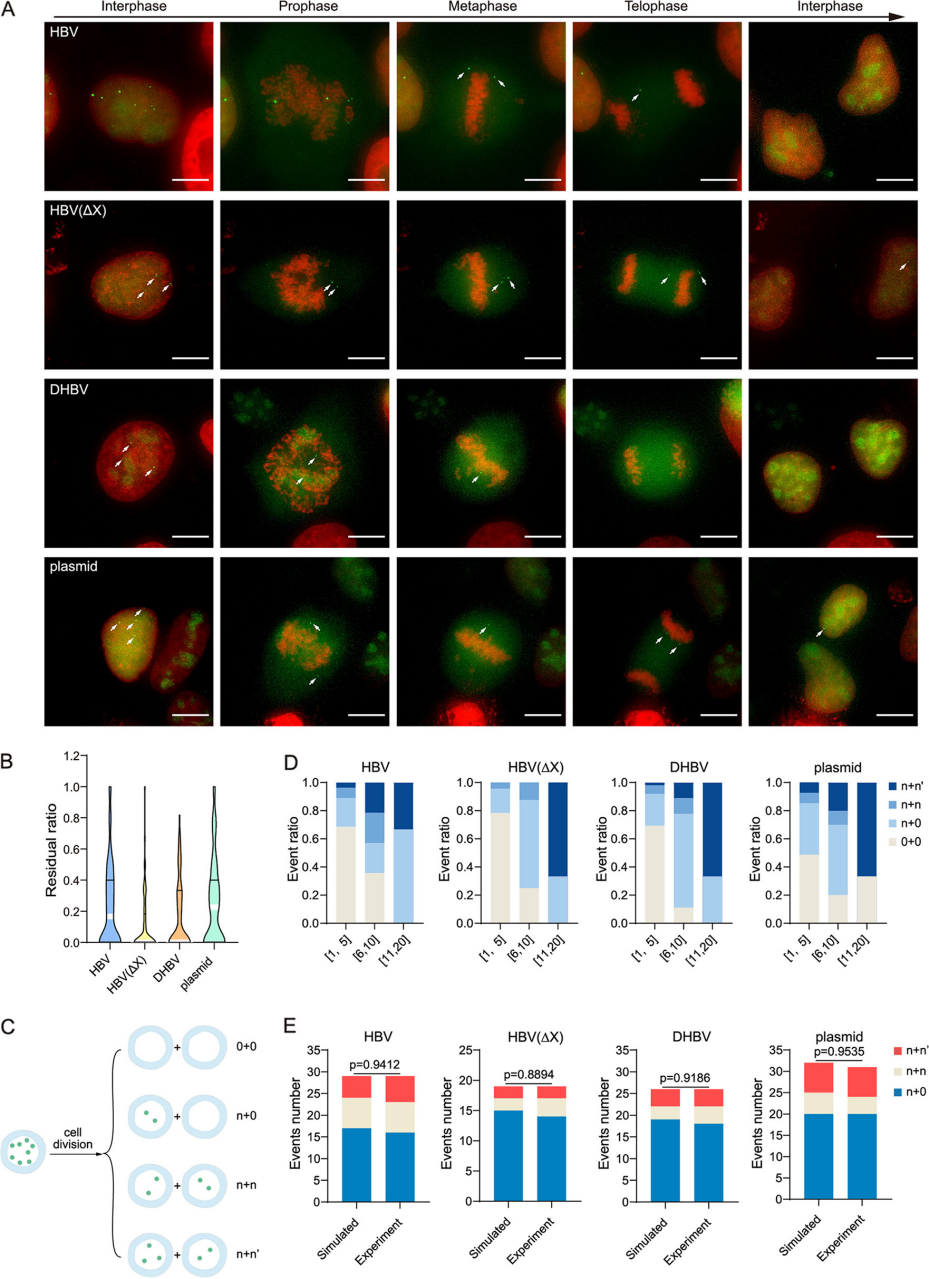
Loss and distribution of HBV rcccDNA–CRISPR-Tag revealed by live-cell imaging. (A) Dynamics of HBV WT (row 1), HBV(ΔX) (row 2), DHBV WT (row 3), and plasmid (row 4) episomes through mitosis are displayed. H2B-mCherry-labeled chromosomes indicate different cell cycle stages. The arrows indicate the episome loci, which were not completely captured during mitosis because the cell thickness exceeds the *z* range. See Movie S3. Bars, 10 μm. (B) The residual ratios of HBV WT (53 events), HBV(ΔX) (57 events), DHBV WT (61 events), and plasmid (54 events) episomes through mitosis were quantified. The white line represents the median, and the black line represents the interquartile range. (C) Schematic illustration for distribution events: completely lost (0+0), distributed to one cell (*n*+0), symmetrically distributed to both cells (*n*+*n*), and asymmetrically distributed to both cells (*n*+*n*′). (D) Event ratios for HBV WT (53 events), HBV(ΔX) (57 events), DHBV WT (61 events), and plasmid (54 events) episomes. (E) Random distribution into two daughter cells of residual loci for viral and prokaryotic episomes. Comparison between simulated and experiment distribution events of HBV WT (29 events), HBV(ΔX) (19 events), DHBV WT (31 events), and prokaryotic (26 events) episomes. *P* values were calculated using a two-sided Fisher’s exact test.

10.1128/mbio.03550-22.3 FIG S3 Construction and characterization of a fluorescent indicator for cell cycle progression (see Fig. 6). (A) Illustration of a fluorescent probe that labels S/G_2_/M-phase nuclei red. (B) The DNA contents of DI-3 and DI-3 expressing mCherry-hGeminin(1–110) were stained with Hoechst 33342 and measured using a flow cytometer. (C) Cell cycle-dependent changes in fluorescence of mCherry-hGeminin(1–110) in DI-3 cells. See Movie S2. Bar, 10 μm. Download FIG S3, TIF file, 2.2 MB. Copyright © 2023 Ding et al. 2023 Ding et al. https://creativecommons.org/licenses/by/4.0/ This content is distributed under the terms of the Creative Commons Attribution 4.0 International license.

10.1128/mbio.03550-22.7 MOVIE S2 Live-cell imaging of cell cycle-dependent changes in fluorescence of mCherry-hGeminin(1–110) (left) and HBV rcccDNA–CRISPR-Tag loci (right) in DI-3 cells (see Fig. S3C). The video was acquired at a single plane. An image was taken every 5 min. *z* projection with a maximum-intensity merge signal is shown. Bar, 10 μm. Download Movie S2, AVI file, 5.2 MB. Copyright © 2023 Ding et al. 2023 Ding et al. https://creativecommons.org/licenses/by/4.0/ This content is distributed under the terms of the Creative Commons Attribution 4.0 International license.

Compared with those of TR–CRISPR-Tag in LANA-expressing cells, focus numbers of rcccDNA were much smaller and did not expand during mitosis, indicating that it cannot replicate in cells. The rcccDNA loci did not attach to cell chromosomes labeled by H2B-mCherry in the metaphase and were substantially lost during mitosis (Fig. 6A, panels 1 and 3; Movie S3). Accompanying quantitative analysis showed that cccDNA molecules were almost completely lost in a low-copy-number setting (1 to 5 copies/cell; medium residual ratio = 0), while a medium of 17% and 20% of cccDNA could be retained with intermediate (6 to 10 copies/cell) and high (11 to 20 copies/cell) initial copy number (Fig. 6B). To explore the pattern of their distribution, we classified distribution events into four distinct types, as follows: completely lost (0+0), distributed to one cell (*n*+0), symmetrically distributed to both cells (*n*+*n*), and asymmetrically distributed to both cells (*n*+*n*′) (Fig. 6C). Results showed that the percentage of distribution types was related to the foci number in mother cells (Fig. 6D). Higher ratios of *n*+*n* and *n*+*n*′ events were related to larger focus numbers in mother cells (Fig. 6D), which suggested a random-distribution pattern. The random distribution was confirmed by a comparison between simulated and experiment data (Fig. 6E). The loss and random-distribution patterns were also observed in prokaryotic plasmid episomes (Fig. 6A, row 4; Fig. 6B, D, and E; Movie S3). As HBx plays a central role in cccDNA transcription and maintenance (34–37, 46), we examined if the dynamics of cccDNA through mitosis was affected by HBx. We found that HBx-null rcccDNAs were substantially lost and distributed randomly to daughter cells after mitosis (Fig. 6A, row 2; Fig. 6B, D, and E; Movie S3).

10.1128/mbio.03550-22.8 MOVIE S3 Live-cell imaging of HBV WT, HBV ΔX, DHBV WT, and plasmid episomes (green) and H2B-mCherry-expressing chromosomes (red) in DI-3 cells undergoing mitosis (see Fig. 6A). The video was acquired as a *z* stack at a 0.5-μm step size and with a total of 20 steps. One *z* stack was taken every 10 min. *z* projection with maximum-intensity merge signal is shown. Bar, 10 μm. Download Movie S3, AVI file, 3.3 MB. Copyright © 2023 Ding et al. 2023 Ding et al. https://creativecommons.org/licenses/by/4.0/ This content is distributed under the terms of the Creative Commons Attribution 4.0 International license.

Overall, above results suggested that HBV and DHBV episomes were substantially lost (~80%) in one-round cell division and randomly distributed to daughter cells.

### Live-cell imaging deciphers CRISPR-tagged cccDNA movements.

**FIG 7:**
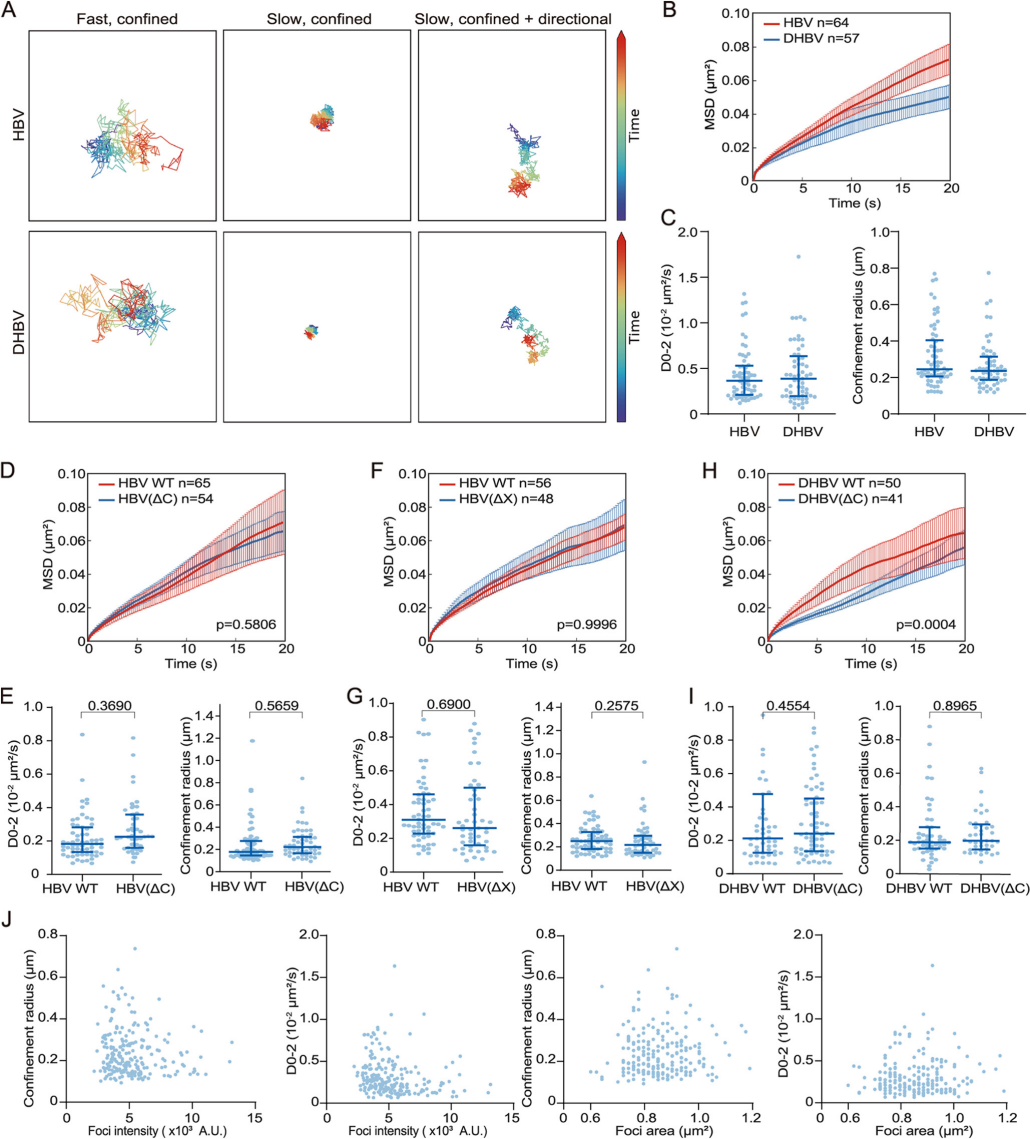
Tracking the movement of rcccDNA–CRISPR-Tag episomes in living cells. (A) Trajectories of HBV and DHBV rcccDNA-CRISPR-Tag episomes with different movement modes (Movie S4). (B) Mean squared displacement (MSD) curves of HBV and DHBV rcccDNA–CRISPR-Tag episomes. (C) Quantification of diffusion coefficient and confinement radius estimated from the MSD curve in panel B. (D, F, and H) MSD curves of HBc-null (D), HBx-null (F), and DHBV HBc-null (H) rcccDNA–CRISPR-Tag episomes. (E, G, and I) Quantification of the diffusion coefficient and confinement radius estimated from the MSD curves in panels (D, F, and H). *P* values were calculated using Kolmogorov-Smirnov test. (J) Scatterplot of rcccDNA focus intensity or area with diffusion coefficient or confinement radius (220 foci were analyzed). MSD curves are displayed as means and standard errors of the means. Bars in graphs show medians and interquartile ranges.

Our live-cell imaging system offers an opportunity to monitor the dynamics of cccDNA in living cells. Hence, we performed high-frequency (0.2 s per frame) time-lapse microscopy to track the movement of CRISPR-tagged cccDNA. Trajectory analysis revealed a confinement movement within a short time (<100 s), occasionally overlaid with slow directed motion (Fig. 7A and B; Movie S4). We measured the diffusion coefficients at 3.644 × 10^−3^ μm^2^/s (median) and 3.872 × 10^−3^ μm^2^/s (median) for HBV and DHBV CRISPR-tagged rcccDNA, respectively (Fig. 7C). These episomes diffused within an area with a 0.25-μm (HBV, median) and a 0.24-μm (DHBV, median) radius (Fig. 7C).

10.1128/mbio.03550-22.9 MOVIE S4 Live-cell imaging of HBV and DHBV rcccDNA–CRISPR-Tag episome movement in DI-3 cells (see Fig. 7A). The videos were acquired at 0.2-s intervals for 500 frames. The trajectories of episomes are displayed. Bar, 5 μm. Download Movie S4, AVI file, 10.9 MB. Copyright © 2023 Ding et al. 2023 Ding et al. https://creativecommons.org/licenses/by/4.0/ This content is distributed under the terms of the Creative Commons Attribution 4.0 International license.

As gene loci at different states have different mobilities (21, 47–50), we tested whether rcccDNA–CRISPR-Tag mobility is related to HBx, HBc, or focus characteristics. The HBx- and HBc-null mutations of HBV and DHBV were validated by the compromised antigen expression, transcription, or replication levels (Fig. 4A–C and Fig. S4). No significant increase of diffusion coefficients and confinement radius caused by the HBx deletion was observed (Fig. 7F and G). Similarly, no significant difference was observed in diffusion coefficients and confinement radius between wild-type and HBc-null rcccDNA episomes (Fig. 7D, E, H, and I). In addition, we did not observe an obvious correlation between focus characteristics and mobility (Fig. 7J). The confinement radius and diffusion coefficient were small for a large percentage of rcccDNA foci (Fig. 7J). The above data suggested that the mobility of dCas9-accessible rcccDNA is not affected by the loss of HBx or HBc. Nevertheless, as HBx inactivation decreased the number of observable rcccDNA (Fig. 4J), an assessment of the dynamics of dCas9-inaccessible molecules is beyond the capability of this system.

## DISCUSSION

HBV cccDNA plays a crucial role in persistent infection, and the failure to eradicate it represents a major obstacle for HBV curative therapy. Understanding the molecular mechanisms affecting the stability of cccDNA could provide new targets for cccDNA clearance. Whereas current studies of cccDNA were almost based on biological purification and bulk measurements, and knowledge concerning the spatiotemporal features of cccDNA were constrained by the paucity of detection methods (8, 9, 11, 51). The present study aimed to develop a novel system to investigate the spatiotemporal dynamics of cccDNA. By leveraging the CRISPR/Cas9-mediated DNA imaging technology and HBV minicircle system, we constructed a cell model to label cccDNA molecules with fluorescent protein, which exhibited high sensitivity and specificity for cccDNA tracking in real time at the single-cell level.

Nevertheless, as the performance of this imaging system is contingent on the binding of sgRNA-loaded dCas9 and the proper recruitment of the GFP1–10 fragment, a number of issues, such as epigenetic modifications, transcriptional activity, nuclear subdomain, and heterochromatin region, will affect the accessibility of rcccDNA to such complexes (Fig. S5). Indeed, a series of cross-validation experiments showed that the number of CRISPR-Tag foci does not totally correlate with the number of active transcribing rcccDNAs or the number of FISH-detectable foci. At low to medium copy numbers, CRISPR-Tag foci exhibited near-perfect colocalization with RNA FISH foci and the intensity of the foci in two fluorescent channels correlated well, suggesting that dCas9 accessibility and transcription activity are nonexclusive. Indeed, using a CRISPR-Tag-derived design of live-cell imaging, Xu et al. reported that a chromatin environment that can facilitate transcriptional activation may cause expanded sizes of dCas9-GFP spots (21). However, at a high copy number, this correlation disappeared. This can also be confirmed by the near absence of cells with >20 CRISPR-Tag foci/cell, whereas RNA FISH and DNA FISH consistently revealed their presence. The reason for the absence of high focus numbers in HBV rcccDNA–CRISPR-Tag imaging is not clear but might be related to the level of dCas9 protein. The prevalent rcccDNA cluster competed with the dCas9 proteins, resulting in the lower signal-to-noise ratio. A cell clone with more appropriate dCas9 expression may solve this problem. Further studies are necessary to explore the reasons and improve the labeling signal-to-noise ratio.

10.1128/mbio.03550-22.4 FIG S4 Validation of HBc-null of DHBV rcccDNA–CRISPR-Tag (see Fig. 7). DI-3 cells were transfected with wild-type (WT) and HBc-null (ΔC) DHBV rcccDNA–CRISPR-Tag. (A) DHBV transcripts were analyzed by Northern blotting. (B) Cytoplasm DHBV core particle-associated DNA was analyzed by qPCR. Data are means and standard deviations. Download FIG S4, TIF file, 0.4 MB. Copyright © 2023 Ding et al. 2023 Ding et al. https://creativecommons.org/licenses/by/4.0/ This content is distributed under the terms of the Creative Commons Attribution 4.0 International license.

10.1128/mbio.03550-22.5 FIG S5 Summary of performance and application of live-cell imaging system for CRISPR-tagged cccDNA. RcccDNA can be labeled with high signal-to-noise ratio when low to medium number of copies are present in the nucleus. CRISPR foci were quantitatively correlated with transcription sites. This live-cell imaging system could be used to study rcccDNA accessibility, fate during cell division, and movement characteristics. Download FIG S5, TIF file, 1.7 MB. Copyright © 2023 Ding et al. 2023 Ding et al. https://creativecommons.org/licenses/by/4.0/ This content is distributed under the terms of the Creative Commons Attribution 4.0 International license.

Another factor related to the accessibility of dCas9 to rcccDNA is the possible presence of subdomains and euchromatin/heterochromatin regions within the nucleus. Indeed, *de novo* formation of the ND10/PML-like intranuclear domains which colocalized with the incoming herpes simplex virus genome has been documented (52, 53). Moreover, the virus-encoded immediate early ICP0 protein was found to be responsible for dispersing these bodies within 2 h after infection (52, 53). Similarly, research on HBx showed that its target, the Smc5/6 complex, was also located in the ND10 bodies and was degraded early after infection. Also, depletion of PML and Sp100, which are part of the ND10 bodies, stimulated the transcription of HBx-null virus in primary human hepatocytes (54). Although no prior reports showed a close spatial relationship between HBV cccDNA and ND10 bodies, it is tempting to speculate that HBx modulates the transcription of cccDNA by influencing its intranuclear location (Fig. S5). Indeed, we found that the number of rcccDNA foci detected by CRISPR-Tag imaging decreased after disruption of HBx, although its actual copy number was unchanged. It is reasonable to postulate that the HBx is important to place cccDNA in an open and dCas9-accessisble state (Fig. S5). This is in agreement with our previous report showing that cccDNA is less susceptible to CRISPR/Cas9-mediated excision when HBx expression is disabled (38). In this regard, our system can then serve as a easy-to-use model for the study of cccDNA accessibility, transcription, and related epigenetic mechanism.

Using our system, this study provided new insights into the fate of HBV and DHBV rcccDNA during cell division. Previous studies reported that cell proliferation induced cccDNA loss and distribution to daughter cells in animal model (13–16, 55). Visualized cccDNA data can avoid interferences from other cccDNA pool maintenance elements in animal models, for example cccDNA formation (11), turnover (10), and degradation (56). In addition, the distribution pattern reported by previous studies were based on mathematical model which was sensitive to assumptions (16). We reported a random-distribution pattern of surviving cccDNA molecules to two daughter cells (Fig. 6), which was based on visualization data and independent of a mathematical model. The substantial loss and random-distribution pattern of cccDNA can result in cccDNA-free daughter cells, confirming that mitosis can result in uninfected liver cells (14). Altogether, the predominant cccDNA destabilization after mitosis suggests a better chance of a cure if virus-positive cells are targeted by an antigen-specific immune response with concomitant hepatocyte renewal. In addition, therapeutic approaches aiming at preventing HBV reinfection and/or maximal quenching of residual viral DNA synthesis and nuclear recycling may further speed up the dissipation of the cccDNA reservoir.

The characteristics of cccDNA locomotion, including motion type, diffusion coefficients, and confinement radius, are similar to gene loci on the chromatin (47–50, 57). As rcccDNA–CRISPR-Tag is much smaller than chromatin, its mobility should theoretically be higher (47, 48). Our observed mobility of rcccDNA is similar to that of chromatin, suggesting that rcccDNA–CRISPR-Tag may associate with immobile structures (47, 48), such as chromatins (9, 58–60). Another factor leading to the reduced mobility is the higher occupation by transcription machinery and hence more nascent RNA in the TS (Fig. 3B), as well as more dCas9-14xGFP binding (Fig. 3B and 4). However, we did not observe a negative correlation between mobility and intensity (Fig. 7J), which argues against this possibility.

We went on to test the effects of HBc and HBx disruption on the mobility of rcccDNA. HBc-null changed neither the mobility nor the accessibility of cccDNA; this indicated that HBc has no noticeable effect on epigenetic regulation of cccDNA, as was also shown by Zhong et al. (61). However, the lack of effect on cccDNA mobility by HBx inactivation (Fig. 7F) should be interpreted with caution. Since the observable rcccDNAs are dCas9 accessible, it is unclear whether the inaccessible ones have similar locomotion characteristics.

Our system has a number of limitations. First, as the CRISPR-Tag is spliced out in pregenomic RNA (pgRNA), we cannot observe newly synthesized cccDNA after intracellular recycling, which precludes the study of these steps. As the presence of CRISPR-Tag in pgRNA disturbs reverse transcription and subsequent intracellular recycling, we need to explore and apply other live-cell labeling strategies to further study the intracellular recycling process. Second, we are also unable to track every cccDNA molecule within the nucleus due to dCas9 accessibility, high copy numbers, and other factors. The observation of the fate of cccDNA during mitosis is restricted to dCas9-accessible molecules. Although it is possible that the cccDNA in the closed state might survive mitosis better, most evidence obtained by means of bulk measurement does not support this idea. Our group also showed that HBV cccDNAs with different accessibility showed similar reduction rates during cell division (38). Third, we cannot rule out the possibility that the foci are the result of a cluster of cccDNA. The irregularity in shape compared with MUC4 foci (single molecule for gene loci on chromosomes) further suggested the cluster of cccDNA molecules in foci. More advanced superresolution imaging techniques may further break diffraction limits and clarify this subject. Further improvements in the imaging model design are necessary to achieve single-molecule cccDNA imaging. Nevertheless, when combined with RNA/DNA FISH or fluorescent tagging of key cellular factors, the CRISPR-Tag recombinant cccDNA imaging system can provide in-depth information to unravel the many unexplored aspects of cccDNA within the 3D landscape of the human chromosome.

## MATERIALS AND METHODS

### Plasmids and DNA imaging cell clones.

Construction of plasmids and cell clones is described in the supplemental material.

### Minicircle DNA production.

The minicircle DNA was produced as reported by Kay et al. (24) with minor modifications. The detailed procedure is described in the supplemental material.

### HBV antigen and nucleic acid quantification.

The HBsAg and HBeAg in the supernatant were measured by enzyme-linked immunosorbent assay (ELISA) kits (Kehua Biotech) according to the manufacturer’s instructions. HBV DNA in the supernatant was examined by qPCR using an HBV DNA quantification kit (Sansure).

### Viral nucleic acid extraction and detection.

Core particle DNA was extracted and detected by Southern blotting as described previously (28). Total RNA was extracted using TRIzol reagent and examined by Northern blotting or qPCR as described previously (28).

### ChIP assay and Western blot assay.

ChIP and Western blot assays were performed as described previously (38). The antibodies used in this study are detailed in Text S1. ChIP DNA was determined by qPCR and high-throughput sequencing.

10.1128/mbio.03550-22.10 TEXT S1 Supplemental materials and methods. Download Text S1, DOCX file, 0.04 MB. Copyright © 2023 Ding et al. 2023 Ding et al. https://creativecommons.org/licenses/by/4.0/ This content is distributed under the terms of the Creative Commons Attribution 4.0 International license.

### FISH.

For DNA FISH to detect nuclear HBV DNA, we followed the method described by Li et al. (16) to isolate nuclei. Then, we followed the procedure described by Yue et al. (17) with some modifications to detect HBV DNA. The detailed methods are described in the supplemental material.

For nuclear RNA FISH to detect the transcription site of rcccDNA, we designed the probe targeting the CRISPR-Tag sequence and followed the procedure described by Zenklusen et al. (62). The detailed procedure is described in the supplemental material.

The probes used in this study are detailed in Text S1.

### Immunofluorescence staining.

Immunofluorescence assay was performed as described previously (17). The cells were immunostained with anti-HBs antibody (Longisland).

### Fluorescence microscopy.

A DeltaVision deconvolution microscope (Applied Precision/GE) equipped with a 60× 1.42-numerical aperture (NA) oil immersion objective and a Photometrics CollSNAP HQ2 charge-coupled device (CCD) camera was used to track fluorescent foci. The snapshot images were captured as *z* stacks with a 0.3-μm step size and with a total of 35 steps. For tracking foci during mitosis, the videos were acquired as *z* stacks with a 0.5-μm step size and with a total of 20 steps. One *z* stack was taken every 5, 10, or 15 min (annotated in each movie and image sequence) to reduce phototoxicity. For diffusion movement tracking, the videos were acquired at 0.2-s intervals for 500 frames. During image acquisition, cells were maintained at 37°C and 5% CO_2_ in an incubator hood.

### Image data analysis.

Postacquisition, images and videos were deconvolved and maximum projected by the DeltaVision build-in program. Imaris (Bitplane) was used for cell segmentation and CRISPR locus fluorescence intensity extraction. The detailed analysis methods are described in the supplemental material.

### Statistical analysis.

Statistical significance was determined by the chi-square test, Kolmogorov-Smirnov test, and Student's *t* test as indicated. *P* values of <0.05 were considered significant.
