# Supplementary figures and images for: Illuminating the Live-Cell Dynamics of Hepatitis B Virus Covalently Closed Circular DNA Using the CRISPR-Tag System
Source: mBio. 2023 Feb 22;14(2):e03550-22. doi: 10.1128/mbio.03550-22 (PMC10128046; doi:10.1128/mbio.03550-22)

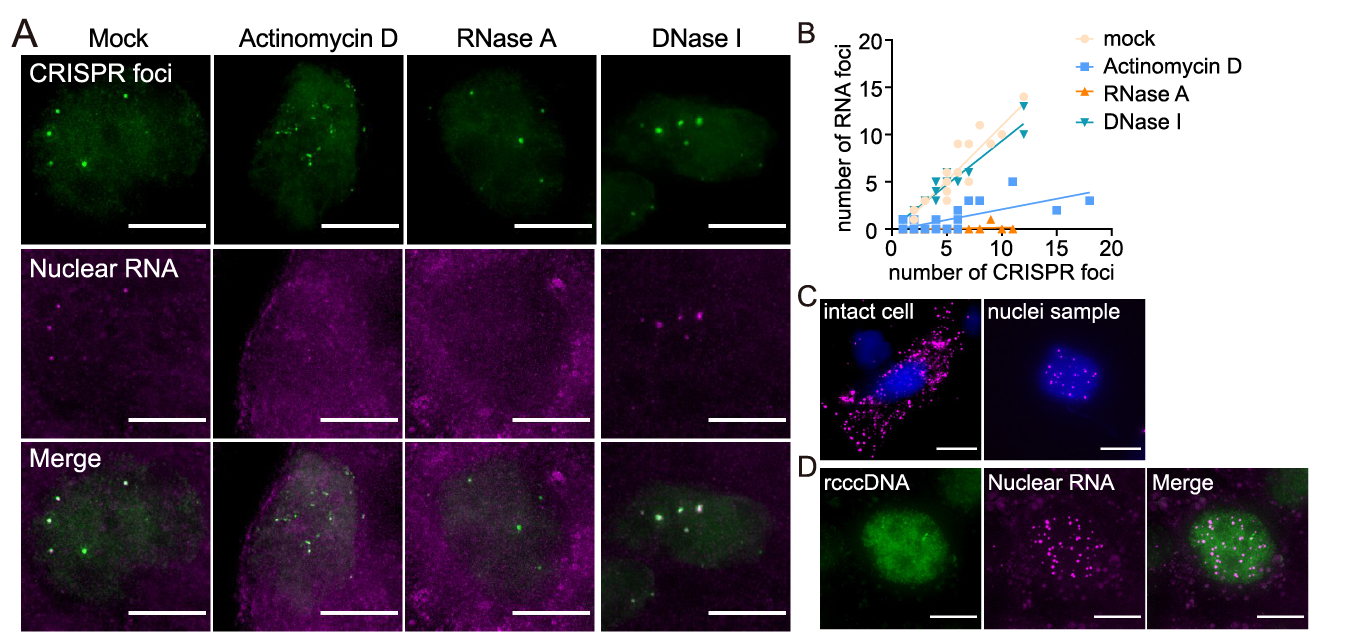

Supplement: FIG S1 [file mbio.03550-22-s0001.tif]

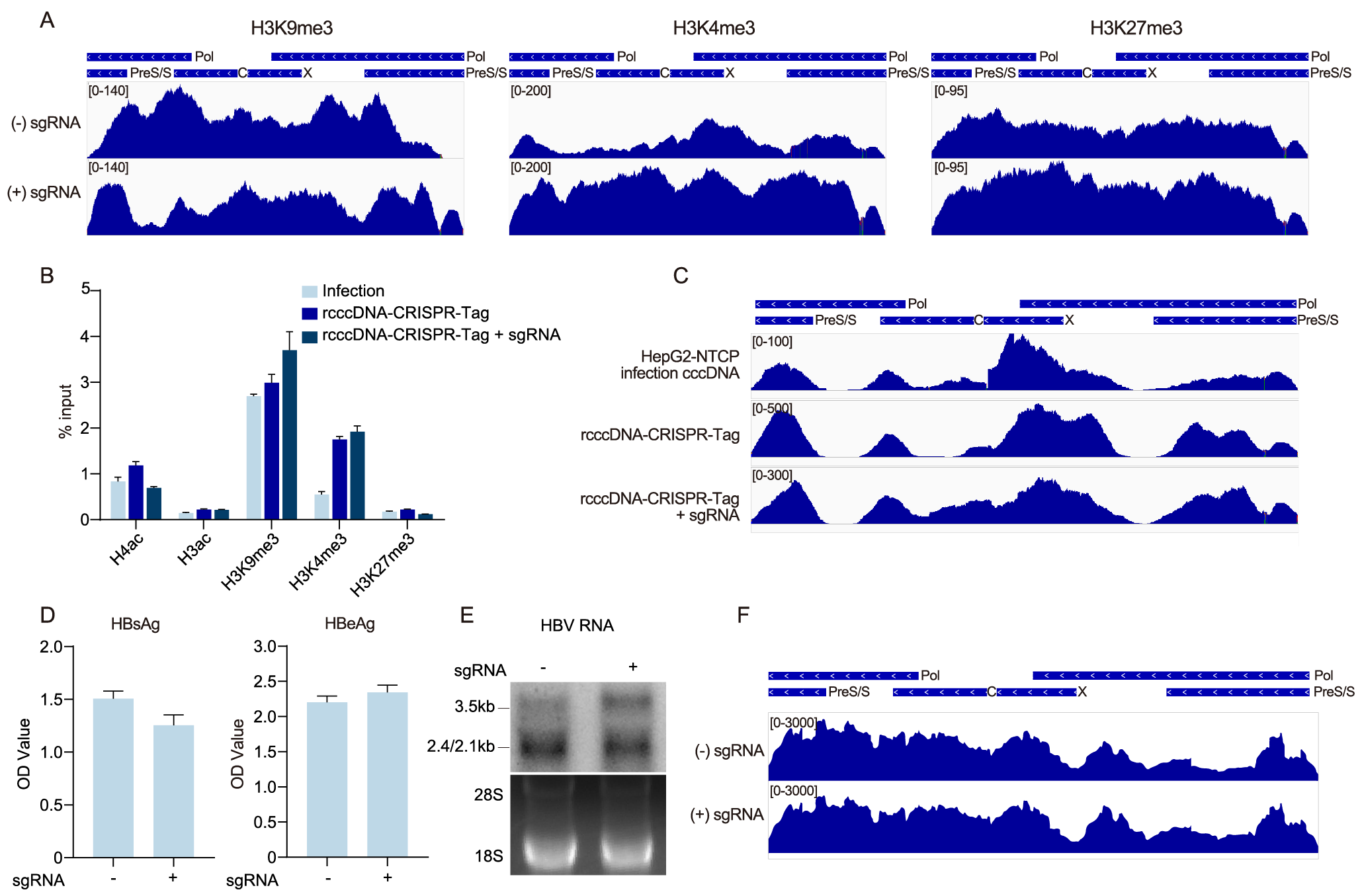

Supplement: FIG S2 [file mbio.03550-22-s0002.tif]

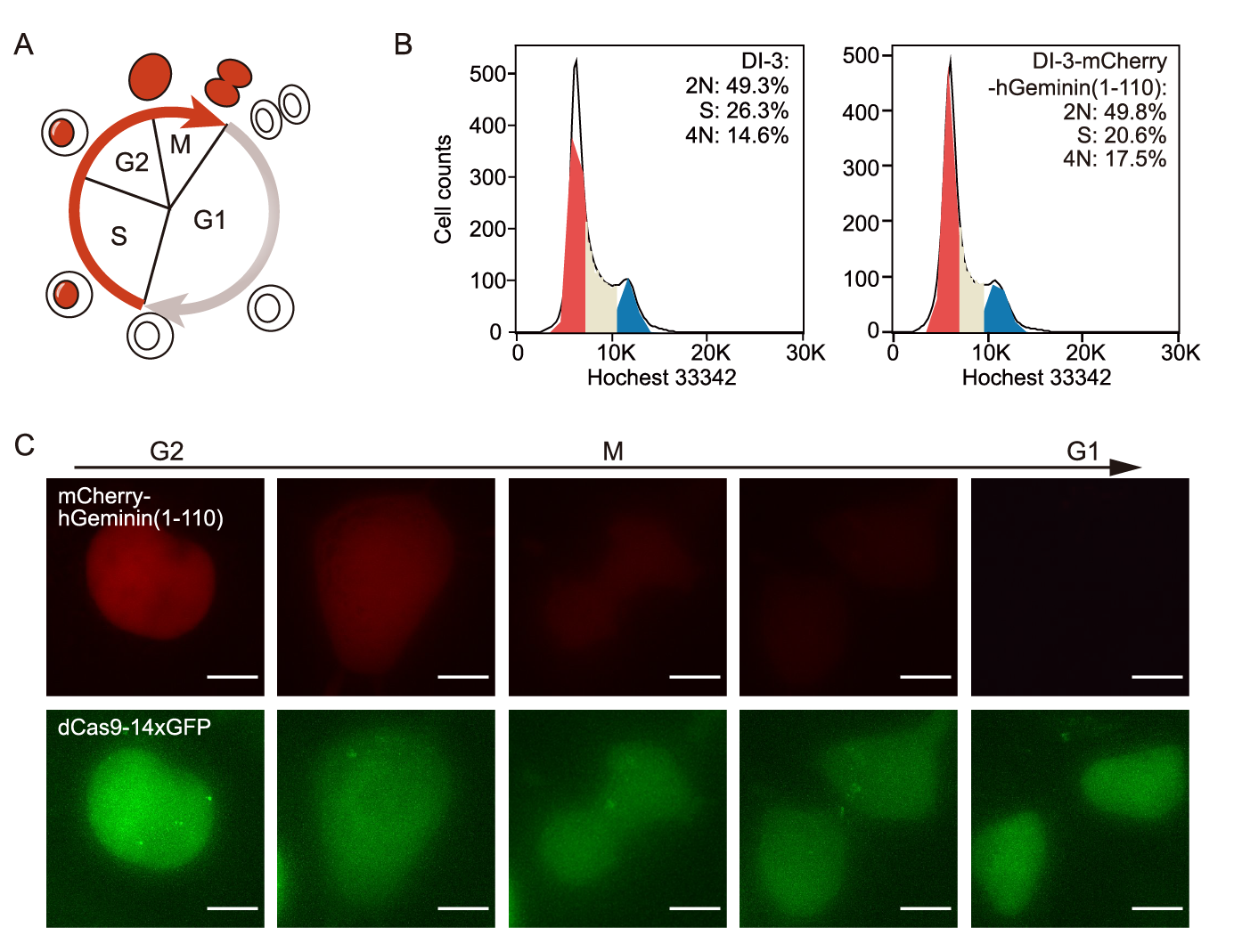

Supplement: FIG S3 [file mbio.03550-22-s0003.tif]

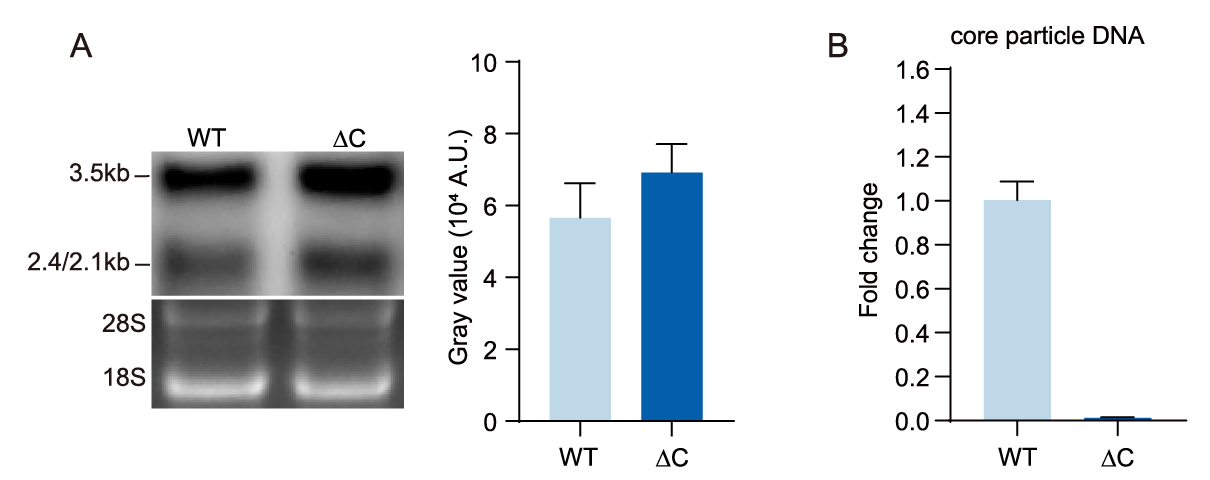

Supplement: FIG S4 [file mbio.03550-22-s0004.tif]

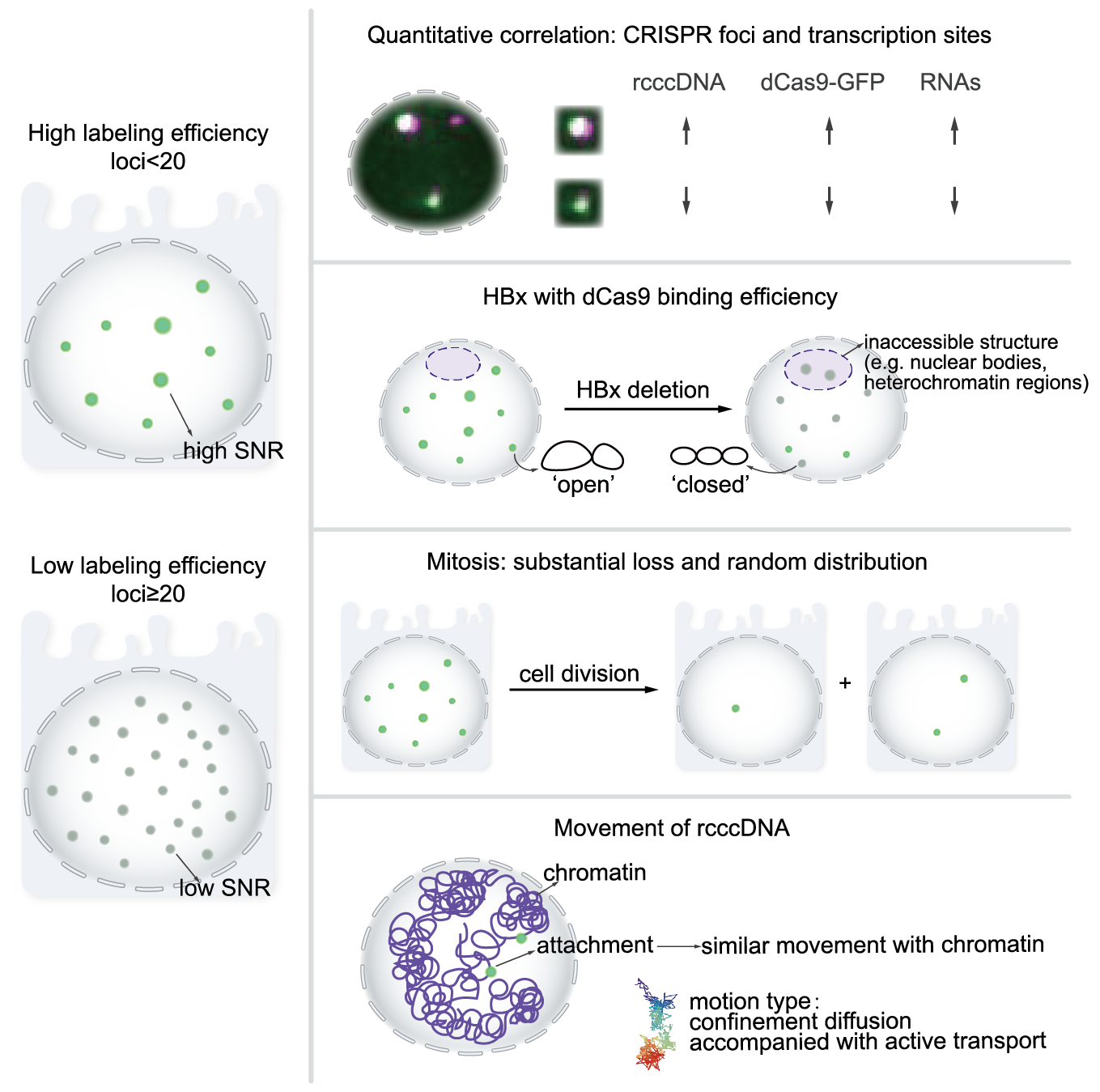

Supplement: FIG S5 [file mbio.03550-22-s0005.tif]
