# Supplementary material for: Illuminating the Live-Cell Dynamics of Hepatitis B Virus Covalently Closed Circular DNA Using the CRISPR-Tag System
Source: mBio. 2023 Feb 22;14(2):e03550-22. doi: 10.1128/mbio.03550-22 (PMC10128046; doi:10.1128/mbio.03550-22)
Supplement: TEXT S1 [file mbio.03550-22-s0010.docx]

***Text S1: Supplemental materials and methods***

**Plasmids**

To construct plasmids for recombinant HBV cccDNA production, monomeric HBV sequence (GenBank accession no. V01460.1) flanked by splicing donor (SD) site and splicing acceptor (SA) site was amplified from pSBi-rcccDNA (1) and cloned into pTubb3-MC (addgene#87112) replacing the EGFP coding sequence. The resulting plasmid was named pMC-HBV. The NotI and SdaI sites were introduced between SA site and attB site to facilitate CRISPR-Tag insertion. The CRISPR-Tag sequence was excised from pMa-CRISPR-Tag_V2 (a kind gift from Prof. Baohui Chen) and subcloned into NotI/SdaI site to generate pMC-HBV-CRISPR-Tag (Fig. 1B, left). A point mutation (C1773T) was introduced by fusion PCR for a premature translation termination codon to construct HBx deletion plasmid. The resulting plasmid was named pMC-HBV(ΔX)-CRISPR-Tag. Two point mutations (A1699G, T1700C) were introduced by fusion PCR to turn start codon (Met) to Ala, which stopped translation initiation of core protein. The resulting plasmid was named pMC-HBV(ΔC)-CRISPR-Tag.

To construct parental plasmid for recombinant DHBV cccDNA production, we assembled four fragments, including DHBV(1413-2540) and DHBV(2517-1412) amplified from pCMV-DHBV (2) (GenBank accession no. K01834), attB-SA and SD-attP amplified from pMC-HBV, into pTubb3-MC (addgene#87112) to construct pMC-DHBV. Using above-mentioned CRISPR-Tag insertion strategy, we obtained pMC-DHBV-CRISPR-Tag. Two point mutations (A2647G, T248C) were introduced by fusion PCR to turn start codon (Met) to Ala, which stopped translation initiation of core protein. The resulting plasmid was named pMC-DHBV(ΔC)-CRISPR-Tag.

For plasmid-CRISPR-Tag, a plasmid of the same size with HBV rcccDNA-CRISPR-Tag only containing plasmid backbone and CRISPR-Tag, a 1529 bp plasmid backbone fragment was amplified from pMC-HBV and inserted between the BglII and MluI sites in pMa-CRISPR-Tag_V2 (a gift from Prof. Baohui Chen) which resulted in a 4009 bp-length plasmid.

To build pTR-CRISPR-Tag that harbors KSHV TR sequence, NotI site was introduced at plasmid-CRISPR-Tag for TR sequence insertion. The TR sequence was excised from pGFP-3TR (a gift from Prof. Qiliang Cai) and subcloned into plasmid-CRISPR-Tag NotI/Eco52I site that resulted in a 4031 bp-length plasmid.

To generate pljm1-mCherry-LANA-3xFlag, mCherry coding sequence amplified from pSLQ1661-sgMUC4-E3 (F+E) (addgene #51025) was subcloned into pljm1-Puromycin vector to replace GFP coding sequence. The LANA-3xFlag was excised from pLVX-YFP-LANA-3xFlag (a gift from Dr. Qiliang Cai) and subcloned into the C-terminal of mCherry to generate pljm1-mCherry-LANA-3xFlag.

To build pljm1-mCherry-hGeminin (1-110), hGeminin was cloned from cDNA library of HepG2 cells and amplified. Overlap extension PCR followed by fragment ligation was used to insert mCherry and hGeminin (1-110) fragments into pljm1-Hygromycin B. The resulting plasmid was named pljm1-mCherry-hGeminin (1-110).

To build pljm1-H2Bj-mCherry, H2Bj was cloned from cDNA library of HepG2 cells and amplified. Overlap extension PCR followed by fragment ligation was used to insert mCherry and H2Bj fragments into pljm1-Puromycin. The resulting plasmid was named pljm1-H2Bj-mCherry.

The CRISPR/Cas9 mediated imaging vector system was kindly provided by Prof. Baohui Chen, including pHR-dSV40-NLS-dCas9-NLS-14xGFP11-NLS_P2A_BFP-NLS, pHR-SFFV-GFP_1-10-NLS, pMa-CRISPR-Tag_V2 (89-90-87)x6 repeats and 3xsgRNAs (89,90,87) for CRISPR-Tag_V2.

Construct pCIdA-HBV1.3, provided from Prof. Jianming Hu, lacks 5′ ɛ RNA signal, which eliminates pregenomic RNA packaging and DNA replication.

Plasmids encoding sgRNA targeted to telomere (Addgene #77046) and MUC4 (Addgene #77047) were purchased from Addgene.

**DNA imaging cell clones**

To generate a cell clone for DNA tracking, lentivirus particles were produced at first. Three plasmids, including VSVG, Gag-Pol and plasmids encoding dCas9-14xGFP11 or GFP1-10-NLS, were transfected to HEK293T cell line using Lipofectamine 3000 (Thermo Fisher Scientific) following the manufacture's recommended protocol. The supernatant containing lentiviruses was harvested 48 h after transfection.

Lentiviruses expressing dCas9-14xGFP11 and GFP1-10-NLS were incubated with HepG2 cells. 48 hours after transduction without antibiotic selection, HpeG2 cells were trypinsized. Five thousand cells were transferred into a 10 cm dish without Poly-L-Lysine (PLL) treatment. We changed medium every 3 days until macroscopic cell colonies formed. We picked the colonies, trypinsized and incubated them until we obtained a monoclonal cell line. To screen a cell clone supporting single-molecule DNA imaging, we seeded the monoclonal cells in 24 well plates with coverslips and transduced lentivirus expressing sgRNA targeting MUC4 gene. Two days after transduction, we fixed the cell and observed them under fluorescent microscope. The cell clones displaying fluorescent foci were labeled and their signal-to-noise ratio (SNR) of MUC4 gene imaging was a criterion to estimate each cell clone. We finally selected two cell clones, clone 1-6 and clone 2-3, displaying fluorescent foci for MUC4 gene and we preserved a clone with better SNR, clone 2-3, and named it DNA imaging cell line 3 (DI-3).

To express mCherry-hGeminin (1-110), mCherry-LANA-3xFlag, or H2Bj-mCherry in DI-3 cells, we incubated lentiviruses encoding mCherry-hGeminin (1-110), mCherry-LANA-3xFlag, or H2Bj-mCherry with DI-3 cells and 48 h after transduction, cells were selected by 100 μg/ml hygromycin B or 5 μg/ml puromycin to generate DI-3 cell line stably expressing mCherry-hGeminin (1-110), mCherry-LANA-3xFlag, or H2Bj-mCherry.

DI-3 cell line, DI-3-mCherry-hGeminin (1-110) cell line, DI-3-mCherry-LANA cell line, DI-3-H2Bj-mCherry cell line, HEK293T cell line and HepG2 cell line were routinely maintained in Dulbecco’s modified Eagle’s medium (DMEM) with high glucose (Corning) supplemented with 10% FBS (Biological Industries) and 25mM HEPES (Gibco).

**Minicircle DNA production**

Parental plasmids, including pMC-HBV, pMC-HBV-CRISPR-Tag, pMC-HBV(ΔX)-CRISPR-Tag, pMC-HBV(ΔC)-CRISPR-Tag, pMC-DHBV, pMC-DHBV-CRISPR-Tag or pMC-DHBV(ΔC)-CRISPR-Tag, were transformed to minicircle producer strain E. coli ZYCY10P3S2T (System Biosciences). Bacteria colony was cultured in 400 ml TB medium at 30°C overnight. Bacteria liquid was added with 400 ml LB medium and L-arabinose (Sigma; final concentration, 0.01% w/v), incubated at 30 °C for 6 hours. Plasmids were extracted and separated by agarose gel. Band located at about 2.0-3.0 kb was cut and purified by gel extraction kit to obtain recombinant cccDNA (HBV rcccDNA, HBV rcccDNA-CRISPR-Tag, HBV(ΔC) rcccDNA-CRISPR-Tag, HBV(ΔX) rcccDNA-CRISPR-Tag, DHBV rcccDNA, DHBV rcccDNA-CRISPR-Tag, DHBV(ΔC) rcccDNA-CRISPR-Tag).

**Micrococcal nuclease high-throughput sequencing**

Micrococcal nuclease high-throughput sequencing (MNase-seq) was performed as described with some modifications (3). Briefly, cells were trypsinized and incubated with 50 U/ml micrococcal nuclease (MNase; NEB) in digestion buffer (10 mM Tris pH 7.4, 15 mM NaCl, 60 mM KCl, 1 mM CaCl_2_, 0.1% Triton X-100, protease inhibitor) at room temperature for 20 min. The reaction was stopped by adding Stop buffer (final concentration: 0.25% SDS, 12.5 mM EDTA, 12.5 mM EGTA). The sample were treated with 150 μg/mL RNase A at 37 °C for 30 min and digested with proteinase K overnight at 56°C. Mononucleosomal DNA was then extracted after gel electrophoresis and applied to high-throughput sequencing.

**Bioinformatics analysis**

Clean data were aligned to HBV sequence (subtype ayw; GenBank accession no. V01460.1) by Bowtie2, and the alignment output files (SAM format) were concerted to BAM files followed by sorting using SAMtools. The resulting file were indexed and visualized using IGV genome browser.

**Preparation of nuclei for FISH**

We followed the method described by Li M et al. (4) to prepare nuclei. Briefly, cells in 6-well plate were trypsinized), suspended in 5 ml of 0.56% KCl, and incubated at room temperature for 10 min. Cells were centrifuged for 5 min at 1,000 rpm. Then cells were fixed twice by suspended by 5 ml of ice-cold methanol-glacial acetic acid (3:1) and cells were centrifuged as above. Then cells were suspended in 1 ml of ice-cold methanol-glacial acetic acid (3:1). Delivering 2 to 3 drops of the suspension onto glass slides positioned over a beaker containing water at a temperature of 65°C, resulting in the retention of nuclei on the glass slides. The slides were then dehydrated by incubation in 70%, 90%, and 100% ethanol for 5 min each at room temperature.

**FISH**

For DNA FISH to detect nuclear HBV DNA, we followed the bDNA-based FISH procedure described by Yue L et al. (5) with some modifications. Briefly, slides with nuclei were rehydrated in 50% ethanol for 5 min, PBS for 10 min at room temperature. Nuclei were digested with DNase I at 37°C for1 h. After washing with PBS 3 times for a total of 15 min, nuclei were incubated in 70% Formamide/2x SSC for 30 min and then heated at 75°C for 2 min. Nuclei slides were incubated with specific probes (100-fold diluted in Probe set diluent QF) at 40°C for 3 h. Washing and signal amplification were performed as described in the ViewRNA assay manual (Thermo Fisher Scientific). Cells were counterstained with Hoechst33342 and finally mounted in fluorescence-antifade mounting medium.

For nuclear RNA FISH to detect transcription site of rcccDNA (6), cells were fixed with 4% paraformaldehyde for 10 min, then incubated with 1% Triton X-100 for 10 min at room temperature. After 2 washes in PBS, cells were permeabilized with 50% ethanol overnight at 4 ℃. Cells were rehydrated for 5 min at room temperature in 2x SSC, 50% Formamide. Cells are hybridized overnight at 37 ℃ in hybridizing solution (Cy5-labeled oligo probe targeted to CRISPR-Tag intron region 2 ng/μl, 10% dextran sulfate, 2 mM vanadyl-ribonucleoside complex, 0.02% RNAse-free BSA, 100 μg/ml salmon sperm DNA, 1 mg/ml Yeast RNA, 2x SSC, 50% Formamide. Cells were washed twice for 30 min at room temperature and finally stained with Hoechst 33342.

The probes used in this study are detailed in Table S2.

**Imaging Data analysis**

Post-acquisition, images and videos were deconvolved and maximum-projected by DeltaVision build-in program. Imaris (Bitplane) was used for cell segmentation and CRISPR loci fluorescent intensity extraction. The detailed analysis methods were described in supplementary material.

***Signal-to-noise ratio*** was calculated as the ratio of the intensity of a fluorescent signal and the power of background noise as following formula:

$$SNR=\frac{P_{signal}}{P_{noise}}=\frac{Max intensity of GFP spot-Mean intensity of background GFP}{Std. dev. of background signal}$$

***Fluorescent foci characteristics*** were analyzed using Imaris (Bitplane). The foci were detected by Imaris. The foci number per nucleus, intensity, and volume were recorded.

***Residual ratio*** during mitosis was calculated as the following formula, the fluorescent foci in the videos were counted manually.

$$residual ratio=\frac{I_{1}+I_{2}}{I_{0}}$$

where *I_0_* is the fluorescent foci number in mother cell, *I_1_* and *I_2_* are the fluorescent foci number in two daughter cells.

***Co-localization*** of LANA and TR-sequence containing episomes was analyzed with plot profile performed using ‘Analyze/Plot Profile’ function, a plugin for ImageJ. The density along the line was then analyzed in Excel and plotted in GraphPad Prism.

***Movement analysis*** was performed using Imaris (Bitplane) and Python. The episome loci movies were deconvolved and maximum-projected by DeltaVision build-in program. Imaris (Bitplane) was utilized to extract the trajectories of episomes loci. Then the trajectories were input to home-build python code for subsequent analysis.

Mean square displacement (MSD) curve as a function of time delay *t=nΔt* was calculated by

$$MSD\left( n\Delta t \right)=\frac{1}{N-1-n}\sum_{i=1}^{N-1-n} \left| r\left( i\Delta t+n\Delta t \right)-r(i\Delta t) \right|^{2}$$

where *Δt* is the frame length (0.2s), *n* is the number of frames (300 frames) in a time delay. *N* is the total number of the frames and *r(t)* is the two-dimensional coordinate (7). For display reasons, all MSD curves data were averaged.

The microscopic diffusion coefficient (D_0-2_ (μm2/s)) was calculated from the slope of the straight line connecting *0-2Δt* using

$$\mathrm{MSD}\left( t \right)=4D_{0-2}t$$

where *t* is the time (8).

Confinement radius (*R*) was calculated by

$$p=\frac{2d}{5}R^{2}$$

where *p* is the plateau of the MSD curve, *d* is the number of dimensions (9).

**References**

1. Wu M, Li J, Yue L, Bai L, Li Y, Chen J, Zhang X, Yuan Z. 2018. Establishment of Cre-mediated HBV recombinant cccDNA (rcccDNA) cell line for cccDNA biology and antiviral screening assays. Antiviral Res 152:45–52.

2. Liu K, Ludgate L, Yuan Z, Hu J. Regulation of Multiple Stages of Hepadnavirus Replication by the Carboxyl-Terminal Domain of Viral Core Protein in trans. J Virol 89:2918–2930.

3. Wang Y, Li Y, Zai W, Hu K, Zhu Y, Deng Q, Wu M, Li Y, Chen J, Yuan Z. HBV covalently closed circular DNA minichromosomes in distinct epigenetic transcriptional states differ in their vulnerability to damage. Hepatology n/a.

4. Li M, Sohn JA, Seeger C. 2018. Distribution of Hepatitis B Virus Nuclear DNA. J Virol 92:e01391-17.

5. Yue L, Li C, Xu M, Wu M, Ding J, Liu J, Zhang X, Yuan Z. 2021. Probing the spatiotemporal patterns of HBV multiplication reveals novel features of its subcellular processes. PLOS Pathog 17:e1009838.

6. Zenklusen D, Larson DR, Singer RH. 2008. Single-RNA counting reveals alternative modes of gene expression in yeast. Nat Struct Mol Biol 15:1263–1271.

7. Chen B, Gilbert LA, Cimini BA, Schnitzbauer J, Zhang W, Li G-W, Park J, Blackburn EH, Weissman JS, Qi LS, Huang B. 2013. Dynamic Imaging of Genomic Loci in Living Human Cells by an Optimized CRISPR/Cas System. Cell 155:1479–1491.

8. Daumas F, Destainville N, Millot C, Lopez A, Dean D, Salomé L. 2003. Confined Diffusion Without Fences of a G-Protein-Coupled Receptor as Revealed by Single Particle Tracking. Biophys J 84:356–366.

9. Dion V, Gasser SM. 2013. Chromatin Movement in the Maintenance of Genome Stability. Cell 152:1355–1364.

**Antibodies used in this study.**

| **Antibodies** | **Cat. NO.** | **Origin** |
| --- | --- | --- |
| Anti-HBs Ab | M-0283 | Long island |
| Anti-H3 Ab | 4620S | Cell signaling |
| Anti-H4 Ab | 14149S | Cell signaling |
| Anti-HA Ab | 3724S | Cell signaling |
| Anti-H3ac Ab | 39139 | Active Motif |
| Anti-H4ac Ab | 39925 | Active Motif |
| Anti-H3K9me3 Ab | 39161 | Active Motif |
| Anti-H3K4me3 Ab | 39915 | Active Motif |
| Anti-H3K27me3 Ab | 39155 | Active Motif |
| Anti-Flag Ab | F1804 | Sigma-Aldrich |
| Anti-β-actin Ab | A2522 | Sigma-Aldrich |

**FISH probes used in this study**

| **Target** | **Sequence** |
| --- | --- |
| CRISPR-Tag-1 | 5’**OH**-CTCGGTATGG**T**GCCCCTCAGGGCC**T**CCGCTTAT**T**CTGACTACTC-3’ |
| CRISPR-Tag-2 | 5’**OH**- CTGCCGCTCG**T**GGTGCCTA**T**GGTAGCTGA**T**GGGCCCCATCA -3’ |
| ViewRNA HBV probe set | PLoS Pathog. 2021 Aug 9;17(8):e1009838. |

The dye modified sites are highlighted.
